# Functional annotation of the cattle genome through systematic discovery and characterization of chromatin states and butyrate-induced variations

**DOI:** 10.1186/s12915-019-0687-8

**Published:** 2019-08-16

**Authors:** Lingzhao Fang, Shuli Liu, Mei Liu, Xiaolong Kang, Shudai Lin, Bingjie Li, Erin E. Connor, Ransom L. Baldwin, Albert Tenesa, Li Ma, George E. Liu, Cong-jun Li

**Affiliations:** 1 Animal Genomics and Improvement Laboratory, BARC, Agricultural Research Service, USDA, Beltsville, MD 20705 USA; 2 Department of Animal and Avian Sciences, University of Maryland, College Park, MD 20742 USA; 3 College of Animal Science and Technology, China Agricultural University, Beijing, 100193 China; 4 College of Animal Science and Technology, Shaanxi Key Laboratory of Agricultural Molecular Biology, Northwest A&F University, Yangling, 712100 Shaanxi China; 5 College of Agriculture, Ningxia University, Yinchuan, 750021 China; 6 Guangdong Provincial Key Lab of Agro-Animal Genomics and Molecular Breeding and Key Lab of Chicken Genetics, Breeding and Reproduction, Ministry of Agriculture, College of Animal Science of South China Agricultural University, Guangzhou, 510642 China; 7 The Roslin Institute, University of Edinburgh, Edinburgh, EH4 2XU UK

**Keywords:** Cattle genome, Functional annotation, Chromatin states, Butyrate, Rumen development

## Abstract

**Background:**

The functional annotation of genomes, including chromatin accessibility and modifications, is important for understanding and effectively utilizing the increased amount of genome sequences reported. However, while such annotation has been well explored in a diverse set of tissues and cell types in human and model organisms, relatively little data are available for livestock genomes, hindering our understanding of complex trait variation, domestication, and adaptive evolution. Here, we present the first complete global landscape of regulatory elements in cattle and explore the dynamics of chromatin states in rumen epithelial cells induced by the rumen developmental regulator—butyrate.

**Results:**

We established the first global map of regulatory elements (15 chromatin states) and defined their coordinated activities in cattle, through genome-wide profiling for six histone modifications, RNA polymerase II, CTCF-binding sites, DNA accessibility, DNA methylation, and transcriptome in rumen epithelial primary cells (REPC), rumen tissues, and Madin-Darby bovine kidney epithelial cells (MDBK). We demonstrated that each chromatin state exhibited specific enrichment for sequence ontology, transcription, methylation, trait-associated variants, gene expression-associated variants, selection signatures, and evolutionarily conserved elements, implying distinct biological functions. After butyrate treatments, we observed that the weak enhancers and flanking active transcriptional start sites (TSS) were the most dynamic chromatin states, occurred concomitantly with significant alterations in gene expression and DNA methylation, which was significantly associated with heifer conception rate and stature economic traits.

**Conclusion:**

Our results demonstrate the crucial role of functional genome annotation for understanding genome regulation, complex trait variation, and adaptive evolution in livestock. Using butyrate to induce the dynamics of the epigenomic landscape, we were able to establish the correlation among nutritional elements, chromatin states, gene activities, and phenotypic outcomes.

**Electronic supplementary material:**

The online version of this article (10.1186/s12915-019-0687-8) contains supplementary material, which is available to authorized users.

## Introduction

Ruminants evolved from simple-stomached animals by transforming into foregut microbial fermenters that could digest grasses and complex carbohydrates [1]. In ruminants, the rumen is central to feed efficiency, methane emission, and productive performance. Rumen microbes digest simple and complex carbohydrates (fiber) and convert them into volatile fatty acids (VFAs; mainly acetic, propionic, and butyric acids), and in fact, VFAs can provide 50 to 70% of a cow’s energy requirements [2]. Interestingly, VFAs not only are nutrients critical to the energy metabolism of the ruminant, but also appear to be responsible for the differentiation during post-natal rumen development [3]. Butyrate has been established as the most potent among VFAs in the induction of changes in cellular functions [4]. Roles for butyrate have been established in the cell differentiation, proliferation, and motility, as well as the induction of cell cycle arrest and apoptosis [5]. Our previous research showed that butyrate can regulate DNA histone modification [6] and gene networks, controlling cellular pathways including cell signaling, proliferation, and apoptosis [7]. In addition, butyrate is a histone deacetylase (HDAC) inhibitor that alters histone acetylation and methylation [8] and, therefore, also functions as an epigenomic regulator [9]. Thus, butyrate-induced biological effects in bovine cells may serve as a paradigm of epigenetic regulation and serve as a model for understanding the full range of butyrate’s potential biological roles and molecular mechanisms in cell growth, proliferation, and energy metabolism [10].

Researchers have discovered a plethora of regulatory elements for controlling genome activities (e.g., gene expression) in human and model organisms, which play central roles in normal development and diseases, hence dramatically improving our biological interpretation of the primary DNA sequence [11–15]. The Roadmap Epigenomics Consortium (2015) defined 15 chromatin states (e.g., promoter/transcript-associated and large-scale repressive states) in humans by combining five histone marks and demonstrated that those states have specific enrichments for DNA methylation and accessibility, as well as for non-exonic evolutionary conserved elements, indicating their distinct biological roles [15]. Kazakevych et al. reported that chromatin states were dramatically changed during the specialization and differentiation of intestinal stem cells in adult humans, suggesting their important roles in normal organ development [16]. In addition to the basic research of genomic biology, having a complete functional annotation of genomes will contribute to understanding the genomic underpinning of complex traits and diseases, thus benefiting precision medicine in humans. For instance, through partitioning heritability of complex traits by different functional annotations, Finucane et al. revealed that the heritability of immunological diseases was highly enriched in FANTOM5 enhancers [17]. Speed and Balding increased the genomic prediction accuracy for complex traits and diseases in both humans and the mouse by differentially weighting genomic variants according to their functional annotations [18].

Although functional annotation of genomes has been well explored in a diverse set of tissues and cell types in human and model organisms, livestock genomes lack such functional annotation. Investigating the global regulatory elements of genomes in livestock not only informs us their basic biology, but also enhances the execution of genomic improvement programs [19, 20]. As shown in previous studies, even with limited functional annotations, investigators could improve QTL detection and genomic prediction for complex traits of economic importance in dairy cattle, particularly in multi-breed scenarios [21–25]. To produce comprehensive maps detailing the functional elements in the genomes of domesticated animal species, a coordinated international effort, the Functional Annotation of Animal Genomes (FAANG) project, was launched in 2015 [26].

**Fig. 1:**
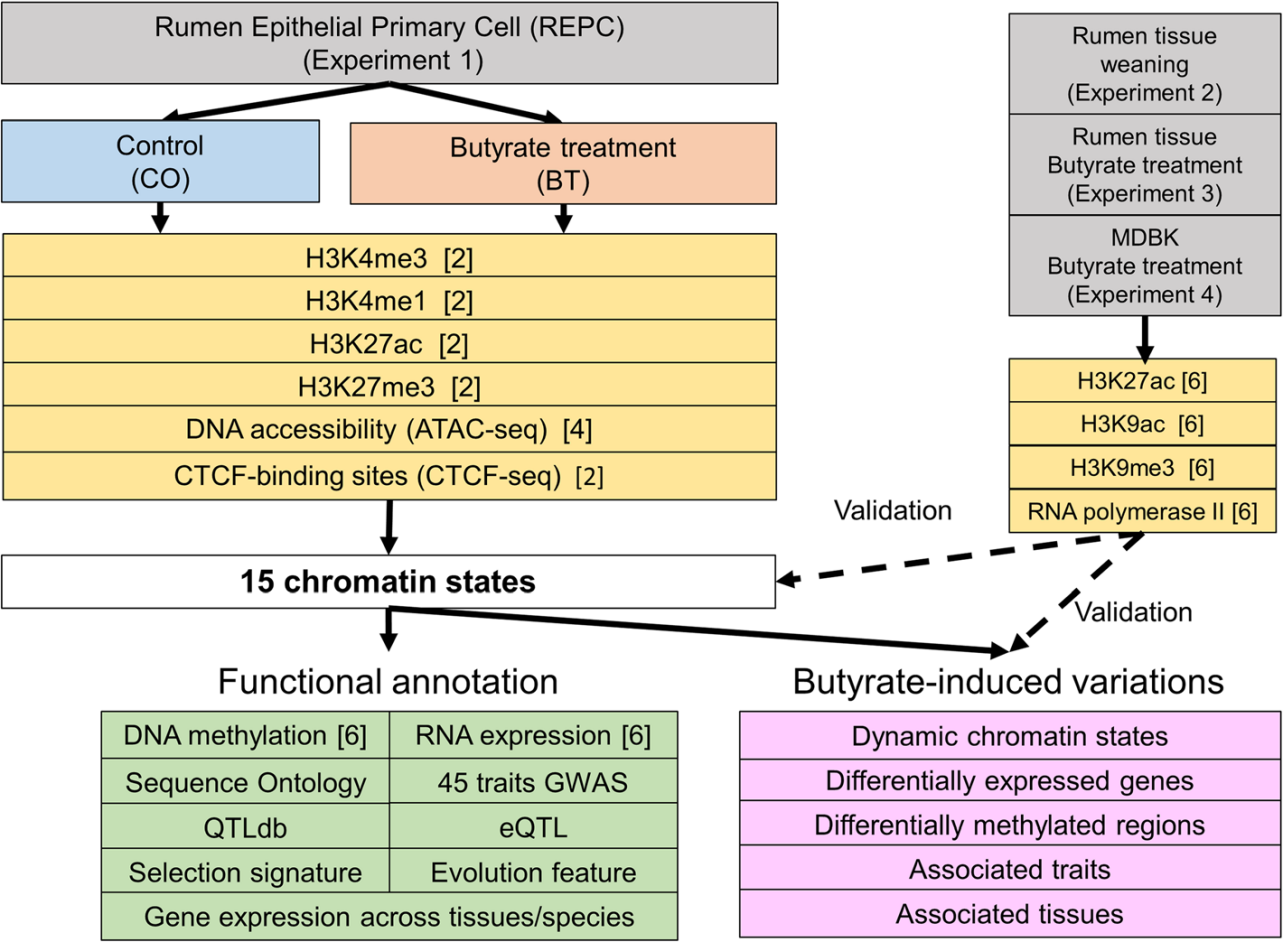
The global study design. Gray boxes represent four distinct studies conducted in rumen epithelial primary cells (REPC) before and after (24 h) butyrate treatment (experiment 1), in rumen tissues before and after weaning (experiment 2), in rumen tissues before and after butyrate treatment (experiment 3), and in Madin-Darby bovine kidney epithelial cells (MDBK) before and after butyrate treatment (experiment 4), respectively. Orange boxes illustrate epigenomic marks generated in each condition per study. Green boxes describe genome-wide data sets used for functional annotation for 15 chromatin states defined in REPC. Pink boxes outline butyrate-induced variations identified in chromatin states, gene expression, and DNA methylation, as well as their potentially affected traits and tissues. Dashed lines denote validation steps we used for findings in REPC by using results generated in experiments 2, 3, and 4. The numbers in the square brackets are the number of assays generated in the current study

To obtain a complete global landscape of regulatory elements in cattle and to explore the dynamics of chromatin states in rumen epithelial cells induced by butyrate (a key regulator for rumen development and an HDAC inhibitor [27]) at early developmental stages, we have conducted the following four experiments (Fig. 1). In the first study, we profiled 26 genome-wide data sets in parallel at high resolution for four histone modifications (i.e., H3K4me3, H3K4me1, H3K27ac, and H3K27me3), DNA accessibility (ATCT-seq), CTCF-binding sites, DNA methylation, and RNA expression in the newly established rumen epithelial primary cells (REPC) before and after (24 h) butyrate treatment, respectively. We then systematically defined and characterized 15 chromatin states by integrating those epigenomic marks with dozens of genome-wide data sets, including sequence ontology, multiple-tissues/species gene expression, DNA methylation, transcription factors, REPC-specific genes, regulatory motif instances, evolutionary conservation elements, large-scale genome-wide association study (GWAS) signals of 45 complex traits, cattle QTLdb, expression quantitative trait loci (eQTLs), and selection signatures in cattle. To understand molecular mechanisms underlying rumen development, we explored the dynamics in chromatin states, DNA methylation, and gene expression, as well as their interplays before and after butyrate treatment. To validate our findings, we conducted another three experiments, where we sequenced three histone modifications (H3K27ac, H3K9ac, and H3K9me3) and RNA polymerase II (RNA poly II) across the entire genome from rumen tissues before and after weaning (experiment 2), before and after butyrate treatment (experiment 3), and in Madin-Darby bovine kidney epithelial cells (MDBK) before and after butyrate treatment (experiment 4), respectively. We verified that the identified chromatin states and butyrate-induced molecular dynamics in REPC were generally consistent in rumen tissues and MDBK. Our study demonstrated the vital role of functional annotation for understanding gene regulation, complex trait variation, domestication, and adaptive evolution in livestock. Our data sets will serve as a valuable resource for interpreting the biological and genetic data sets in cattle, such as GWAS of diverse complex phenotypes, and thereby benefiting their genomic improvements.

## Results

### General characteristics of epigenomic, DNA methylation, and transcriptomic data sets

Among the four experiments, we generated a total of 38 genome-wide epigenomic data sets at a high resolution, including six different histone marks (H3K4me3, H3K4me1, H3K27ac, H3K27me3, H3K9ac, and H3K9me3), RNA poly II, ATAC, and CTCF, producing a total of 1,545,698,388 clean paired-end reads with an average uniquely mapping rate of 73.20%. Additionally, we profiled six RNA-seq data sets and six whole-genome bisulfite sequencing (WGBS) data sets from REPC to explore changes in gene expression and DNA methylation before and after (24 h) butyrate treatment, producing a total of 83,648,115 (the average uniquely mapped rate of 86.9%) and 362,173,297 (31.9%) clean paired-end reads, respectively. Details of summary statistics for all 50 newly generated data sets are described in Additional file 2: Table S1.

For all 38 epigenomic data sets, as shown in Additional file 1: Figure S1a, we obtained a total of 1,624,657 peaks with an average of 42,754 (ranging from 738 for RNA pol II in the rumen tissue before weaning to 187,475 for H3K27ac in MDBK following butyrate treatment). In general, we obtained more peaks from the two cell lines (i.e., REPC and MDBK) than actual rumen tissues, possibly reflecting a sensitivity issue for measuring epigenomic marks in the actual tissues. The corresponding genome coverage for peaks in each sample had an average of 1.31% (ranging from 0.01% for RNA poly II in rumen tissue to 11.87% for H3K27me3 in REPC following butyrate treatment) (Additional file 1: Figure S1b). At 24 h post butyrate treatment in REPC, we observed CTCF, H3K27me3, and H3K4me3 generally increased their genome coverage percentage, whereas H3K27ac, H3K4me1, and ATAC lost their genome coverage percentage (Additional file 1: Figure S1b). We observed that the repressive histone mark, H3K27me3, exhibited a greater peak length than the other epigenomic marks (Additional file 1: Figure S2). These epigenomic marks exhibited a bimodal distribution along with their nearest genes, with one peak overlapped with the corresponding gene body and the other ~ 100 kb away from the gene body (Additional file 1: Figure S3). The first peak agrees with the enrichment of transcriptional start sites (TSS) with epigenomic marks, indicating the existence of *cis*-regulatory mechanisms underlying gene expression [28]. The second peak might imply the existence of long-range regulatory elements (e.g., enhancers and insulators); however, further researches are required for a better understanding of its functional impacts on the gene activities. Both of the two repressive histone marks, H3K27me3 and H3K9me3, exhibited a higher peak at ~ 100 kb away from the gene body compared to the other epigenomic marks (Additional file 1: Figure S3). In addition, we found that correlations of peak-length vs. exon-length were higher than those of peak-length vs. gene-length and peak-length vs. chromosome-length (Additional file 1: Figure S4–S6), indicating the epigenomic peaks were more likely to be associated with exons as compared to genes and chromosomes. This might support that epigenomic marks play important roles in the transcriptional regulation [11, 15]. We also observed that CTCF and ATAC from the REPC sets were associated with many active histone modifications (e.g., H3K4me1, H3K4me3, RNA poly II, H3K9ac, and H3K27ac) in both REPC and rumen tissues (Additional file 1: Figure S7a), demonstrating that epigenomic modification shared certain similarities between the primary cells and rumen tissues. We identified that gene expression correlations of samples within groups (three biological replicates) were very high (*r* > 0.99), with a clear separation between samples from control and butyrate treatment (Additional file 1: Figure S7b). However, DNA methylation correlations among the six samples did not show a clear group-based pattern (Additional file 1: Figure S7c), consistent with the concept that DNA methylation is a relatively long-term regulator of gene expression compared to other epigenomic modifications [29]. This suggests that DNA methylation may not regulate transcriptional changes in a short term, such as tested here for only 24 h after butyrate treatment.

### Systematic definition and characterization of 15 chromatin states in cattle

**Fig. 2:**
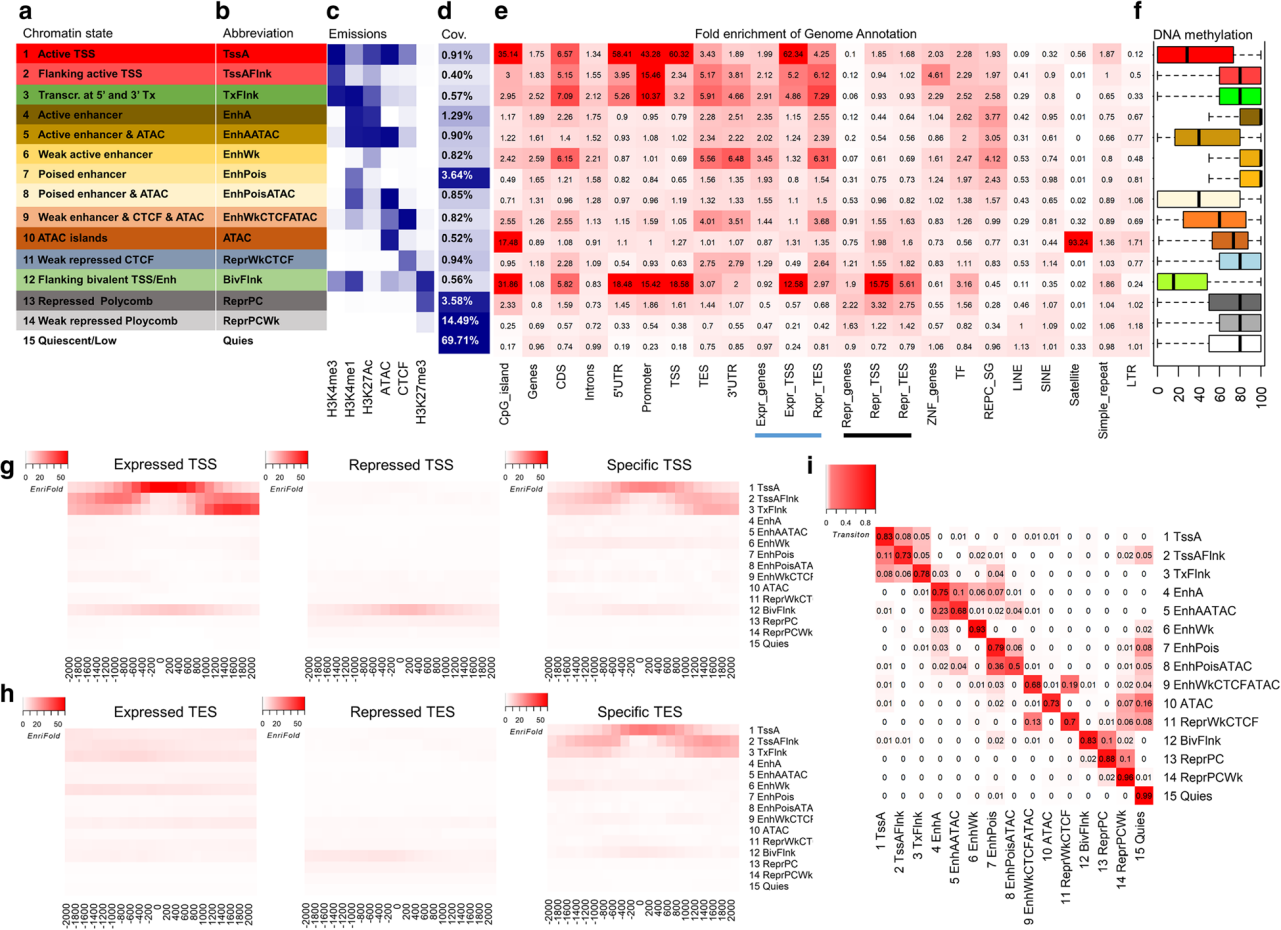
Definition and characteristics of 15 chromatin states in rumen epithelial primary cells (REPC). **a**, **b** Definitions and abbreviations of 15 chromatin states, respectively. **c** Emission probabilities of individual epigenomic marks for each chromatin state. **d** Genomic coverages of chromatin states. **e** Enrichments of chromatin states for diverse genomic annotations, including CpG islands, gene contents (promoters were defined as ± 1 kb around transcription start site, TSS), expressed genes (FPKM > 0, *n* = 14,839) in REPC, repressive genes (FPKM = 0, *n* = 9777) in REPC, transcription factors (TF), genes specifically highly expressed in REPC (REPC_SG, *n* = 1230), and common repeats. **f** DNA methylation across 15 chromatin states in REPC. **g**, **h** Enrichments of chromatin states around ± 2 kb of TSS and TES of expressed genes, repressive genes, and REPC-SG genes, respectively. **i** Probabilities of chromatin state transitions learned from ChromHMM, reflects the proximal genomic locations among chromatin states

The particular combinations of epigenomic marks in a genomic region can have distinct biological functionality, often known as distinct chromatin states [13]. Here, we defined 15 chromatin states along the genome, including elements such as promoter/transcript-, enhancers-, bivalent TSS/enhancers-, and repressive-associated states, through the integration of four histone modifications with ATAC and CTCF data in REPC (Fig. 2a–c). The first three states identified were (1) strongly active promoters/transcripts, indicating active TSS (TssA); (2) flanking active TSS (TssAFlnk); and (3) transcribed at gene 5′ and 3′ (TxFlnk), which were found to cover 1.88% of the entire genome. They were characterized by a high frequency of H3K4me3 in common and high enrichments near promoter regions (± 1 kb around TSS of 24,616 Ensembl genes), protein-coding regions, zinc finger genes, transcription factors [30], and expressed genes (FPKM > 0, *n* = 14,839), but not repressed genes (FPKM = 0, *n* = 9777) (Fig. 2d, e). TssA also exhibits a characteristically high enrichment for CpG islands, corresponding to a low level of DNA methylation (Fig. 2f), thereby enhancing the expression of nearby genes and confirming the well-known negative correlation of promoter methylation and gene expression [31]. Meanwhile, TssAFlnk and TxFlnk exhibited high levels of methylation, again consistent with high DNA methylation levels of gene bodies being positively correlating to gene expression [31]. By further evaluating gene TSS and TES, we observed that the first three states had high enrichment in the neighborhood (± 2 kb) of TSS and TES for expressed genes in REPC, but not for repressed genes (Fig. 2g, h). TssA centered at TSS of expressed genes, while TssAFlnk and TxFlnk flanked around TSS of expressed genes (Fig. 2g, h). The transition parameters (reflecting the proximal genomic locations) among chromatin states learned from ChromHMM suggested that the first three states were more likely to transition among one another rather than to other states, while TssAFlnk was more likely to transition to the quiescent state than TssA and TxFlnk were (Fig. 2i).

We detected genes (*n* = 1230) with specifically high expression in REPC by comparing gene expression of REPC to that of 77 other somatic tissues and cell types from cattle, while excluding similar tissues in the gastrointestinal tract (Additional file 1: Figure S8). We found REPC-specific genes were significantly engaged in oxidation-reduction and metabolic processes (Additional file 1: Figure S8) and more likely to be enriched for active enhancers (chromatin states 4–6: active enhancer, EnhA; active enhancer with ATAC, EnhAATAC; and weak active enhancer, EnhWk) as compared to the other chromatin states (Fig. 2e), indicating the tissue specificity of many enhancers for ensuring tissue-specific gene expression [32]. The neighboring regions of both TSS and TES of REPC-specific genes were enriched for the active promoter/transcript-associated states (chromatin states 1–3) (Fig. 2g, h). We observed that ATAC peaks (chromatin state 10) were highly enriched for CpG islands and satellite DNA, suggesting that chromatin structure of CpG islands and satellite DNA create an accessible environment for RNA polymerase II and other transcriptional components to bind [33]. Of note was the flanking bivalent TSS/enhancers (chromatin state 12, BivFlnk, covering 0.56% of the entire genome), which was not only enriched near TSS of expressed genes but was also enriched near TSS of repressed genes. BivFlnk also had a low level of DNA methylation and had high enrichment for CpG islands, promoter regions, and transcription factors, similar to active promoter/transcript-associated states (Fig. 2d–f). We observed that repressive Polycomb (chromatin state 13, ReprPC, covering 3.58% of the entire genome) exhibited higher enrichment near repressed genes than expressed genes and had a high level of DNA methylation (Fig. 2e, f), indicative of their critical roles in gene repression. The transition parameters among chromatin states learned from ChromHMM suggested that the weak/poised enhancer-associated states and ATAC state were more likely to transition to the quiescent state than any other states (Fig. 2i).

**Fig. 3:**
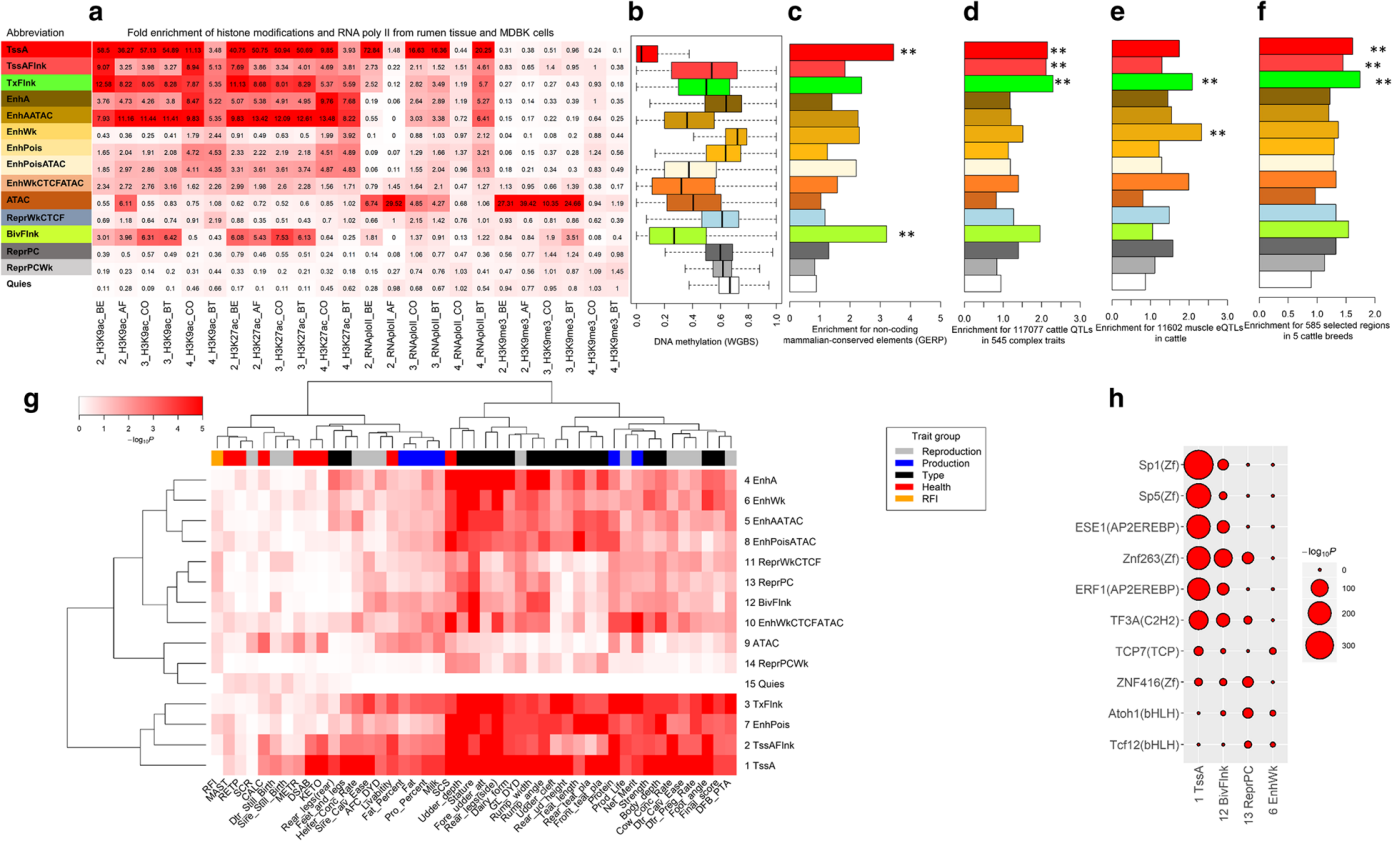
Functional characteristics of 15 chromatin states. **a** Fold of enrichments for epigenomic marks in rumen tissues (experiments 2 and 3) and the MDBK cell line (experiment 4). CO and BT represent the control and butyrate treatment groups, respectively, while BE and AF represent before and after weaning, respectively. **b** DNA methylation patterns of the 15 chromatin states in rumen tissue. **c** Fold of enrichments for non-coding mammalian conserved elements (GERP). **d** Fold of enrichments for 117,077 QTLs (length < 10 kb) of 545 complex traits in cattle QTLdb. **e** Fold of enrichments for 11,602 muscle eQTLs in cattle. **f** Fold of enrichments for 585 selected regions in 5 cattle breeds. The statistical significance for enrichments were calculated using Fisher’s exact test, where “**” means *P* < 0.01. **g** GWAS signal enrichments of 45 complex traits in the US Holstein population. **h** The top five enriched motifs among the four representative chromatin states

By overlapping chromatin states with epigenomic marks in rumen tissues and the MDBK cell line, we validated that chromatin states associated with TssA, TssAFlnk, TxFlnk, EnhA, and EnhAATAC (chromatin states 1–5) were highly over-represented for the two histone marks associated with promoters and enhancers (H3K9ac and H3K27ac). In contrast, these chromatin states were not over-represented for the repressive histone mark (H3K9me3), in both rumen tissues and MDBK (Fig. 3a). We also found TssA profoundly enriched for RNA poly II among rumen tissues and MDBK. Of note, DNA methylation patterns of 15 chromatin states in rumen tissues were highly similar to those in REPC (Figs. 2f and 3b). For instance, TssA was also hypomethylated in rumen tissue (Fig. 3b). We further identified that TssA had the highest enrichment for non-exonic mammalian conserved elements (Fig. 3c). These observations demonstrate the majority of defined chromatin states in REPC were consistent across the tissues and cell types tested [15]. One divergent finding was that the chromatin state BivFlnk only enriched for H3K9ac and H3K27ac in rumen tissues and cells not MDBK, suggesting its possible tissue/cell type specificity (Fig. 3a). Similarly, ATAC state profoundly enriched for RNA poly II and the repressive histone mark, H3K9me3, in rumen tissues but not for MDBK (Fig. 3a). By examining the 117,077 QTLs for 545 complex traits in cattle QTLdb (release 37, Dec. 23, 2018) [34], we confirmed that active promoters/transcripts (chromatin states 1–3), followed by BivFlnk, exhibited the highest enrichment for all these QTLs as compared to the other chromatin states evaluated (Fig. 3d). Because previous studies showed that the majority of eQTLs were conserved across tissues [28, 35], we then overlapped chromatin states with muscle eQTLs in cattle [36] and revealed that weak enhancers (chromatin states 6 and 9) and TxFlnk had the highest enrichment for eQTLs among all 15 chromatin states (Fig. 3e). We also demonstrated that active promoters/transcripts had the highest enrichment for selection signatures that were detected in five cattle breeds in our previous study [37] (Fig. 3f), demonstrating that positive selection is more likely to be associated with active promoters and transcripts.

Our large-scale GWAS signal enrichment analysis revealed that active promoters and transcripts (i.e., TssA, TssAFlnk, and TxFlnk) were the top enriched chromatin states across 45 complex traits of economic importance in the US Holstein population (Fig. 3g), in line with the findings in cattle QTLdb (Fig. 3d). Interestingly, enhancer-associated regions (e.g., EnhA, EnhWk, EnhAATAC, and EnhPoisATAC), which were likely to be tissue specific, were specifically enriched for body type traits (particularly for stature) and somatic cell score (an indicator of mastitis resistance), suggesting the potential roles of rumen epithelial cells in growth and innate immune responses (Fig. 3g). The motif enrichment analysis revealed that 136 out of 922 tested motifs were significantly (adjusted *P* < 0.01) enriched in TssA, mainly including motif families of zinc finger (*n* = 21), AP2EREBP (*n* = 40), and C2C2dof (*n* = 20) (Additional file 3: Table S2). This observation demonstrates that TssA is a hotspot for transcription regulatory factors, and implies that highly expressed genes also require a complex regulatory mechanism to ensure their proper function. We found that BivFlnk enriched for similar motifs as TssA, whereas ReprPC and EnhWk enriched for distinct motifs, such as Atoh1 and Tcf12, which belong to the bHLH family (Fig. 3h).

**Fig. 4:**
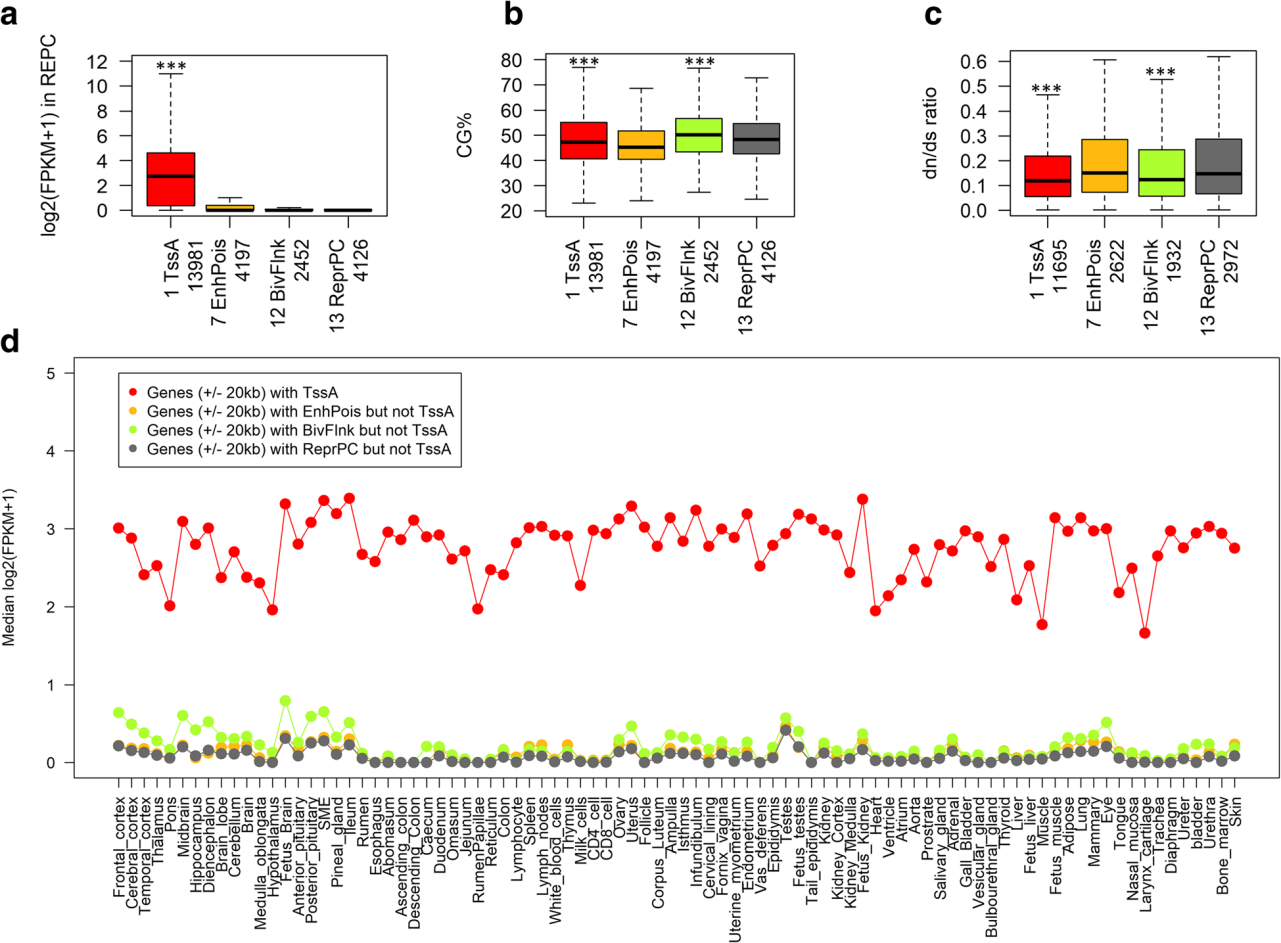
Characteristics of four gene sets with distinct chromatin states. Genes with active promoters (TssA; *n* = 13,981), genes with poised enhancers but not active promoters (EnhPois; *n* = 4197), genes with flanking bivalent TSS/enhance but not active promoters (BivFlnk; *n* = 2452), and genes with repressive Polycomb but not active promoters (ReprPC; *n* = 4126). **a**, **b** Comparisons of expression and CG percentages among the four gene sets, respectively. **c** The dn/ds ratio comparison for the four gene sets corresponding to human-cattle orthologous genes. The statistical significances for comparisons were calculated using *t* test, where “***” means *P* < 0.001. **d** The expression (median of log_2_ (FPKM+ 1)) for the four gene sets across 88 somatic tissues and cell types in cattle

To explore relationships between chromatin states and gene expression, we classified genes into four categories with distinct chromatin states, including (1) genes (*n* = 13,981) with TssA (TssA-genes), (2) genes (*n* = 4197) with poised enhancers (chromatin state 7, EnhPois) but not TssA (EnhPois-genes), (3) genes (*n* = 2452) with BivFlnk but not TssA (BivFlnk-genes), and (4) genes (*n* = 4126) with ReprPC but not TssA (ReprPC-genes). We found that TssA-genes had the highest expression in REPC, followed by EnhPois-genes (Fig. 4a). We also observed that TssA-genes and BivFlnk-genes had a higher CG density and a greater gene-length than EnhPois-genes and ReprPC-genes (Fig. 4b; Additional file 1: Figure S9). By examining dn/ds ratios of orthologous genes (protein evolution) in human vs. cattle, mouse vs. cattle, dog vs. cattle, pig vs. cattle, and sheep vs. cattle, we found that TssA-genes and BivFlnk-genes were also consistently constrained evolutionarily compared to the other two gene sets (Fig. 4c; Additional file 1: Figure S10). We observed that TssA-genes were consistently highly expressed among 89 somatic tissues and cell types in cattle, indicative of the conservation of TssA among tissues and cell types, whereas BivFlnk-genes tended to have a higher expression in brain regions compared to other tissues and cell types (Fig. 4d), indicating a probable regulatory connection between the brain and the digestive system [38]. We further confirmed that orthologues of TssA-genes were conservatively expressed at high levels among 53, 159, and 174 major tissues in human, mouse, and sheep, respectively (Additional file 1: Figure S11–S13). Functional enrichment analysis identified that TssA-genes were significantly engaged in basic cellular processes, including the peptide biosynthetic process, translation, and RNA and enzyme binding, as well as main function in the nucleolus (Additional file 1: Figure S14a-d). In contrast, the remaining three groups of genes were significantly involved in the signaling receptor and hormone activities, and organismal development, as well as function at the extracellular space (Additional file 1: Figure S14a-d). These findings further indicate that the chromatin state of active promoters is evolutionarily conserved at both DNA sequence and gene expression levels, which is consistent with our previous results demonstrating methylation patterns in the promoters of orthologous genes in sperm were generally conserved across mammals [25].

### Butyrate-induced changes in chromatin states, gene expression, and DNA methylation

**Fig. 5:**
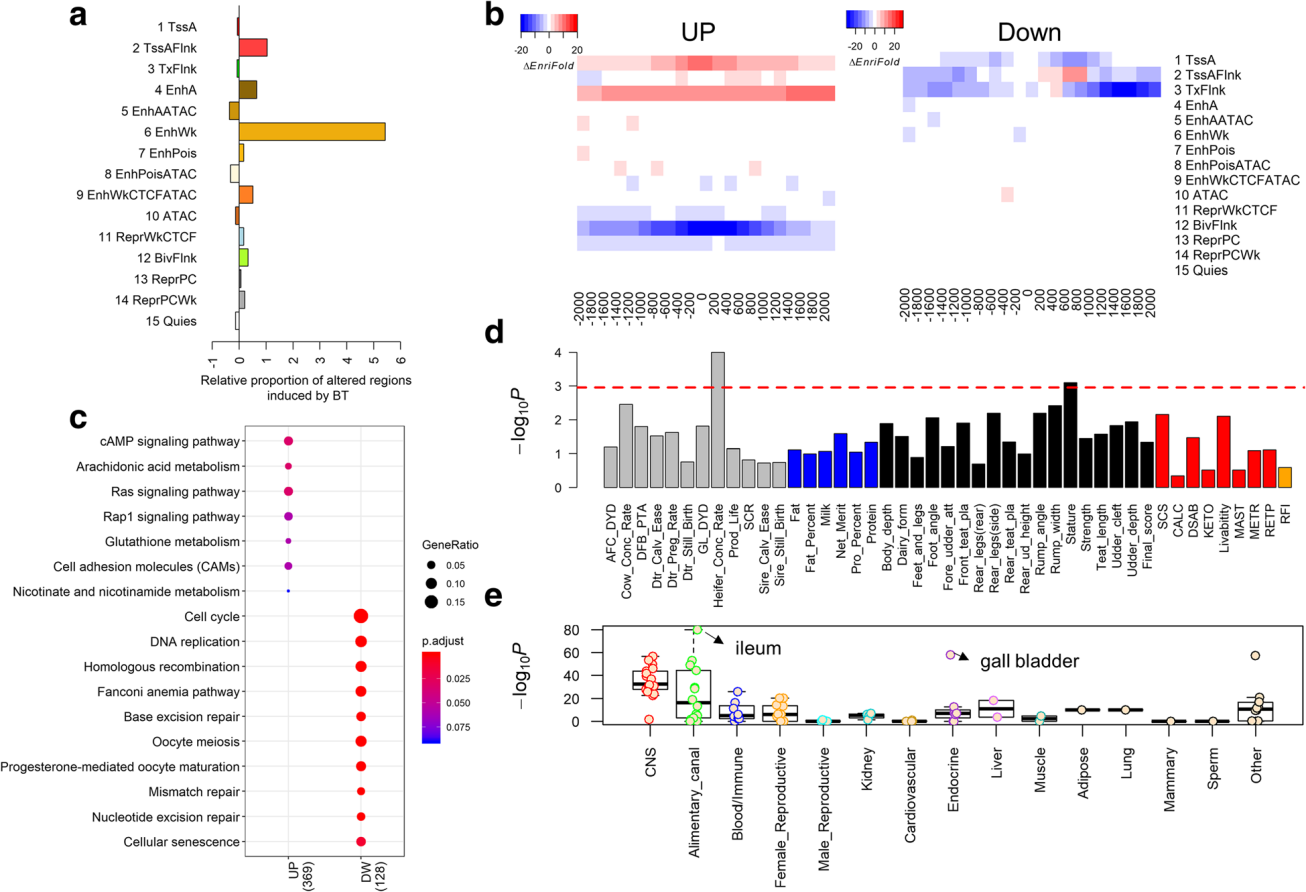
Butyrate-induced dynamics in chromatin states, gene expression, and their associated traits and tissues. **a** Relative proportion of changed regions induced by butyrate treatment (24 h) in rumen epithelial primary cells (REPC). The relative proportion of changed regions for a chromatin state was calculated as the altered (increased or decreased) length of this chromatin state during butyrate treatment divided by the total length of this particular chromatin state before treatment. **b** Changes of enrichment folds of upregulated (left) and downregulated (right) differentially expressed genes (DEGs) for 15 chromatin states before and after butyrate treatment, respectively. **c** Significantly enriched KEGG pathways for up- and downregulated DEGs, respectively. **d** GWAS signal enrichments of DEGs for 45 complex traits in cattle. The red dashed line corresponds to Bonferroni-corrected *P* value = 0.05. **e** Tissue-specific gene enrichment analysis (hypergeometric test) for DEGs

The four histone marks, CTCF, and ATAC of butyrate-treated REPC were assayed as a vital step towards a comprehensive understanding of the molecular mechanism of butyrate-induced genome activities [39]. After 24-h treatment of REPC with 5 mM butyrate in the media, we observed the greatest changes in chromatin states for the weak enhancer and TssAFlnk states, which showed 6.43- and 2.04-fold increases in their overall proportion of regions as compared to the control group, respectively (Fig. 5a). In total, we detected 1266 differentially expressed genes (DEGs) induced by butyrate treatment, including 934 up- and 332 downregulated DEGs, respectively (Additional file 4: Table S3 and Additional file 5: Table S4). Interestingly, we found that TSS of upregulated DEGs (± 20 kb) acquired enrichments for TssA and TxFlnk, while losing enrichment for BivFlnk and ReprPC following butyrate exposure, demonstrating that a portion of BivFlnk likely transitioned into active promoters/transcripts post butyrate treatment, and thereby increased the net expression of the corresponding genes (Fig. 5b). The TSS of downregulated DEGs decreased TssA, TssAFlnk, and TxFlnk enrichments likely explaining the concomitant reduction in their gene expression (Fig. 5b). These findings demonstrate the crucial interplay between chromatin states and gene expression in rumen epithelial cells during butyrate exposure. Functional enrichment analysis further illustrated that upregulated DEGs were engaged in the cAMP signaling pathway, arachidonic acid metabolism, and Ras signaling pathway, while downregulated DEGs were involved in the cell cycle, DNA replication, and oocyte meiosis (Fig. 5c). Interestingly, GWAS signal enrichment analysis demonstrated that these DEGs were also significantly associated with economic traits in dairy cattle, like heifer conception rate and stature (Fig. 5d). Tissue-specific gene enrichment analysis further revealed that these DEGs were highly expressed not only in the digestive system (e.g., ileum and duodenum) but also in the brain regions (e.g., hippocampus and frontal cortex) (Fig. 5e; Additional file 6: Table S5), providing putative evidence for the existence of a gut-brain axis, possibly due to direct or indirect interaction between enteric microbiota and the central nervous system [40]. Although the vast majority of DNA methylation was retained during butyrate treatment, the total of 40 differentially methylated regions (DMRs) exhibited the highest enrichment for ATAC and BivFlnk states (Additional file 1: Figure S15; Additional file 7: Table S6).

**Fig. 6:**
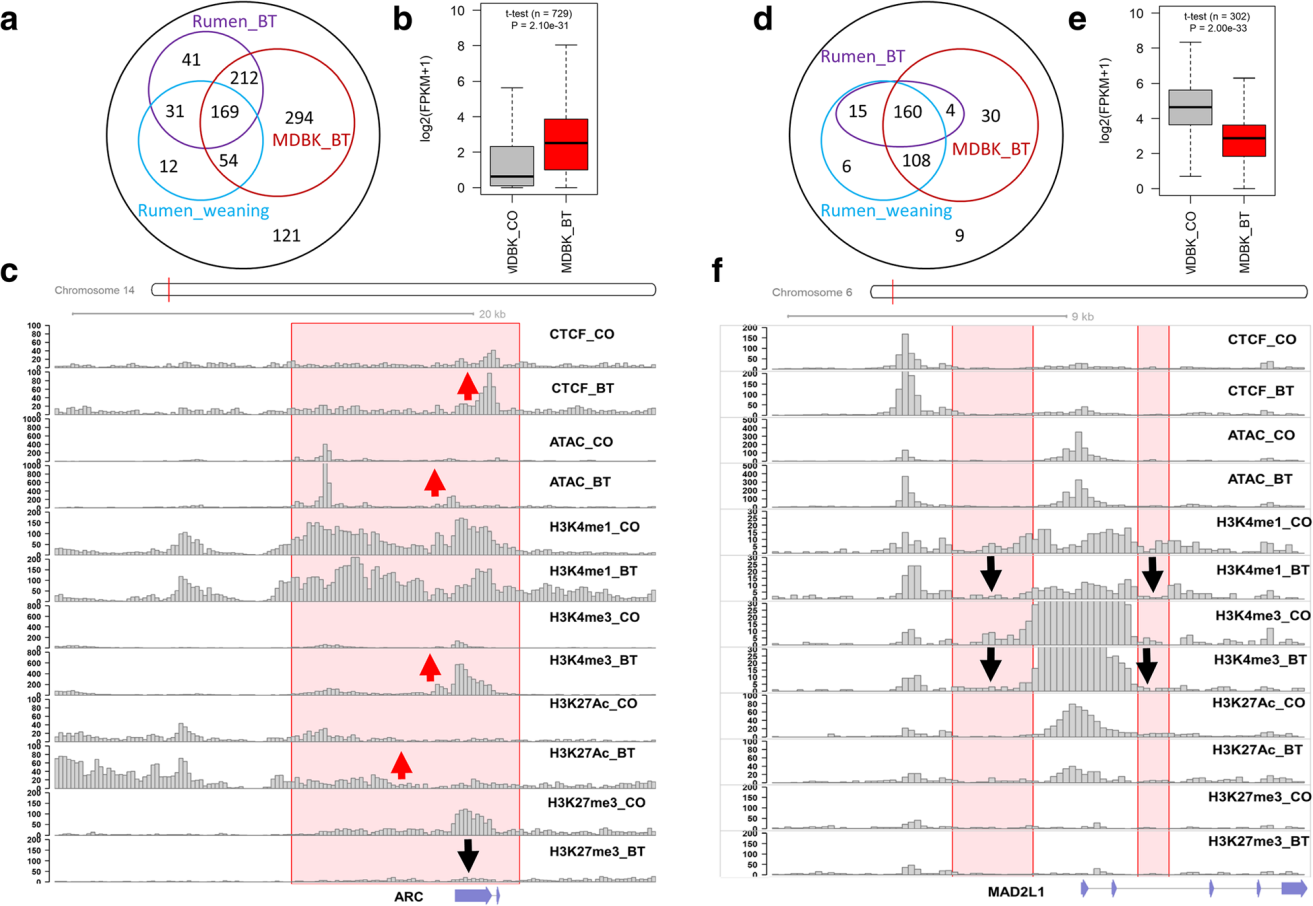
Comparisons of differentially expressed genes (DEGs) and alterations of chromatin states in REPC, rumen tissues, and MDBK. **a** Overlaps of upregulated DEGs post butyrate treatment in REPC with genes gaining at least one of three active epigenomic marks (H3K9ac, H3K27ac, and RNA pol II) after butyrate treatment or weaning in rumen tissues or after butyrate treatment in MDBK, respectively. **b** Comparison of expression for 729 upregulated DEGs also gaining active epigenomic marks in MDBK before and after butyrate treatment. **c** Changes of individual epigenomic marks of *ARC* gene before and after butyrate treatment in REPC, shown as an example of upregulated DEGs that have the chromatin state transition from BivFlnk to active promoter/transcript (highlighted region). The bars are read-counts of the input epigenomic sequence within each 200-bp window. **d** Overlaps of downregulated DEGs post butyrate treatment in REPC with genes losing at least one of three active epigenomic marks (H3K9ac, H3K27ac, and RNA pol II) after butyrate treatment or weaning in rumen tissues or after butyrate treatment in MDBK, respectively. **e** Comparison of expression for 302 downregulated DEGs also losing active epigenomic marks in MDBK before and after butyrate treatment. **f** Changes of individual epigenomic marks of *MAD2L1* gene before and after butyrate treatment in REPC, shown as an example of downregulated DEGs exhibiting the chromatin state transition from TssAFlnk to quiescence (Quies) (highlighted regions)

On a genome-wide basis, we observed 61.41% of BivFlnk was retained after 24-h butyrate treatments as compared to the control group, while ~ 20% transitioned to active promoter/transcript states (the first three chromatin states), indicating upregulation of the corresponding genes (Additional file 1: Figure S16a). Noticeably, we found 470 out of 934 upregulated DEGs (± 20 kb) were associated with the transition from BivFlnk to active promoter/transcript states (TssA, TaaAFlnk, and TxFlnk) at 24 h post butyrate treatment, and fold changes of these genes were significantly greater than the other upregulated DEGs (Additional file 1: Figure S16b). The remaining upregulated DEGs were more likely to gain the chromatin state associated with the weak enhancer, followed by BivFlnk and active enhancer (Additional file 1: Figure S16c). In addition, we found that 266, 453, and 729 out of the 934 upregulated DEGs gained at least one of the three active epigenomic marks (i.e., H3K9ac, H3K27ac, and RNA pol II) in the rumen tissue after weaning, in the rumen tissue after butyrate treatment, and in MDBK after butyrate treatment, respectively (Fig. 6a). By examining the transcriptome data in MDBK before and after butyrate treatment [41], we confirmed that expression levels of those 729 genes were also significantly upregulated at 24 h post butyrate treatment (Fig. 6b), indicating that butyrate might induce similar cellular responses across different cell types and tissues. We showed one example—*ARC* gene which plays key roles in the regulation of both synaptic plasticity and immune system [42, 43] (fold change = 23.26) in Fig. 6c as an example of upregulated DEGs whose chromatin state transitioned from BivFlnk to TssA, TssAFlnk, and TxFlnk after butyrate treatment.

Among the first three active chromatin states, we observed that TssA was more stable during butyrate treatment, as 76.03% retained, while only 59.94% and 43.19% of TssAFlnk and TxFlnk were retained, respectively. Of note was TssAFlnk, which transitioned 11.31% to the quiescent state, whereas only 0.07% and 0.54% transitioned for TssA and TxFlnk, respectively (Additional file 1: Figure S17a). Within the 332 downregulated DEGs (± 20Kb), we found the top five most dynamic chromatin states induced by butyrate treatment were transitions from TssAFlnk and TxFlnk to the weak enhancer, quiescent, active enhancer, and poised enhancer (Additional file 1: Figure S17a). We found that 289, 179, and 302 out of the 332 downregulated DEGs (± 20Kb) also exhibited a loss of at least one of the three active epigenomic marks (i.e., H3K9ac, H3K27ac, and RNA pol II) in the rumen tissues after weaning, in the rumen tissues with butyrate treatment, and in MDBK with butyrate treatment, respectively (Fig. 6d). By examining the transcriptome from MDBK cell responses before and after butyrate treatment, we verified that expression of 302 out of 332 genes was significantly downregulated at 24 h with butyrate treatment (Fig. 6e). We showed changes of individual epigenomic marks of *MAD2L1* gene (fold change = − 27.54) before and after butyrate treatment in Fig. 6f, as an example of the downregulated DEGs. *MAD2L1* is a key component of the mitotic spindle assembly checkpoint and associates with multiple tumor processes [44, 45].

## Discussion

In summary, we established the first global map of regulatory elements (15 unique chromatin states) and defined their coordinated activities in cattle, through genome-wide profiling for six specific histone modifications, RNA polymerase II, CTCF-binding sites, DNA accessibility, DNA methylation, and transcriptomes in rumen epithelial primary cells (REPC), rumen tissues, and Madin-Darby bovine kidney epithelial cells (MDBK). Functional annotations of genome in the REPC capture a remarkable diversity of genomic functions encoded by distinct chromatin states and show that a majority of them are consistent across tissues and cell types. We identified significant associations of chromatin states with gene expression and DNA methylation, as well as demonstrated the importance of comprehensive functional annotation to facilitating the improved understanding of the genetic basis underpinning complex trait variation, eQTLs, positive selection, and adaptive evolution in cattle. Our findings directly support the concept that proximal regulatory elements contribute to positive selection and adaptive evolution of modern sheep breeds, while a previous study reported a similar idea through cross-species mapping of human functional annotation data on to the sheep genome [51]. Additionally, we observed that a large proportion (~ 70%) of the cattle genome of rumen REPC exists in a quiescent state, similar to findings from human tissues where approximately two thirds of the reference epigenome in each tissue and cell type are quiescent [15, 52].

Ruminant species utilize VFAs as their major nutrient energy resources [3]. Most of the VFAs are uptaken and utilized in the rumen epithelium and other gastrointestinal organs [2]. The intrinsic necessities of VFAs add a level of increased sensitivity to ruminant cells. The full range of the biological roles and the molecular mechanisms that butyrate may play in bovine genomic activities has been intensively studied in vitro and in vivo. At 5-mM concentration, butyrate induces specific changes of gene expression and epigenomic landscapes in MDBK cells [5–7, 10, 41]. Comparing to the MDBK cell line, REPC provides a better in vitro model and mimic the rumen epithelium much closely than MDBK cells. To validate the data from in vitro experiment with REPC, in vivo experiments with the rumen tissues before and after weaning and rumen tissues before and after butyrate treatment by direct infusion [53] were also performed with ChIP sequencing. Our data suggested that the majority of defined chromatin states in REPC were generally consistent across tissues and cell types. Certainly, future studies with additional epigenomic marks and tissues/cell types are required for a more comprehensive functional annotation of the cattle genome and validation of the essential roles of butyrate played in rumen development and genetic activities.

Furthermore, our data provided strong verification that butyrate can change the epigenomic landscapes and chromatin states in both rumen tissues and cell lines, resulting in specific changes in gene expression and influencing rumen differentiation/development. We illustrated that the up- and downregulated genes induced by butyrate treatment exhibited distinctive variations in chromatin states and altered biological functions. It has been generally accepted that histone modifications play a crucial role in controlling gene expression. Butyrate, as a native HDAC inhibitor, re-induces histone post-translational modifications and, thus, regulates cell growth, apoptosis, and cell differentiation in many types of cancer [46]. Many previously published reports were dedicated to the biological effects of butyrate on cancer cells. As a result, there is a wealth of knowledge on butyrate as an HDAC inhibitor, the role of aberrant histone acetylation in tumorigenesis, and the potential for cancer chemoprevention and therapy [46–49]. There is little, if any, information about the biological impacts of butyrate in “normal” cells. And there is even less literature available addressing the fundamental mechanism of epigenomic regulatory activities of butyrate in rumen development and function. The HDAC inhibition activity of butyrate makes it a uniquely suited inducer for specific changes in the epigenomic landscape in the foregut of ruminants. Delineating the extent to which the epigenomic landscape and chromatin states are modified by butyrate-induced histone post-translational modification is a critical step in the path to understanding how this nutrient is perturbing specific transcriptomes at the mechanistic level. By surveying butyrate-induced dynamic variation of chromatin states concomitantly with changes in transcription activities observed in REPC, for the first time, we were able to establish strong correlations between nutritional elements, histone modifications, chromatin states, genomic activities, and cellular functions in cattle. Our findings also shed light on the putative use of HDAC functionality in chemoprevention therapies for malignant and non-malignant, hyperproliferative, and inflammatory disorders in humans [50].

## Conclusions

We established the first global map of regulatory elements (15 chromatin states) and defined their coordinated activities in cattle. By integrating a range of genome-wide data sets, such as multiple-tissues/species gene expression, DNA methylation, trait-associated variants, selection signatures, and evolutionary conservation elements, we demonstrate the crucial role of functional genome annotation for understanding genome regulation, complex trait variation, and adaptive evolution in livestock. Using butyrate to induce the dynamics of the epigenomic landscape, we observed the correlation among nutritional elements, chromatin states, gene activities, and phenotypic outcomes.

## Methods

### Sample collections and next-generation sequencing

In the current study, all animal procedures were conducted under the approval of the Beltsville Agricultural Research Center (BARC) Institutional Animal Care Protocol Number 15-008. Animal experimental procedures (butyrate infusion and rumen biopsies), RNA extraction, and sequencing were detailed in our previous report [53]. Rumen primary epithelial cells were isolated from a 2-week-old Holstein bull calf fed with milk replacer only. The methods for rumen epithelial cell isolation and culture were reported previously [54]. The MDBK cell line was purchased from ATCC (ATCC CCL-22; Manassas, VA, USA) and grown in Eagle’s essential medium with 5% fetal bovine serum.

#### Butyrate treatment of cell culture

Ruminant species have evolved to metabolize the short-chain fatty acids to fulfill up to 70% of their nutrient energy requirements [2, 55]. The concentration of short-chain fatty acids in ruminant species is much higher than that in humans and other animals [2]. Based on our previous experiment with MDBK cells, treatment of 5 mM butyrate in vitro can induce significant changes in histone acetylation level and transcription activities without induced significant apoptosis [6]. Thus, 5 mM butyrate was added to the culture medium for 24 h for butyrate treatment of cells.

ATAC-seq, CTCF-seq, and ChIP-seq of H3K27ac, H3K27m3, H3K4m1, and H3K4m3 in rumen primary epithelial cells (RPEC) were performed by using NextSeq 500 (Illumina, Inc. San Diego, CA, USA) at Active Motif, Inc. (Carlsbad, CA, USA). ChIP-seq of rumen epithelial tissues and MDBK cells was performed as reported in our earlier publication [10]. In short, DNA recovered from a conventional ChIP procedure was quantified using the QuantiFluor fluorometer (Promega, Madison, WI, USA). The DNA integrity was verified using the Agilent Bioanalyzer 2100 (Agilent; Palo Alto, CA, USA). The DNA was then processed, including end repair, adaptor ligation, and size selection, using an Illumina sample prep kit following the manufacturer’s instructions (Illumina, Inc., San Diego, CA, USA). Final DNA libraries were validated and sequenced at 75-nt per sequence read, using an Illumina HiSeq 2500 platform.

#### RNA extraction and RNA sequencing

RNA extraction was following the procedure reported previously [41]. Total RNA from six rumen epithelial cell samples was extracted using Trizol (Invitrogen, Carlsbad, CA, USA) followed by DNase digestion and Qiagen RNeasy column purification (Qiagen, Valencia, CA, USA). The RNA integrity was verified using Agilent Bioanalyzer 2100 (Agilent, Palo Alto, CA, USA). High-quality RNA (RNA integrity number [RIN]: 9.0) was processed using an Illumina TruSeq RNA sample prep kit following the manufacturer’s instruction (Illumina, Inc., San Diego, CA, USA). After quality control (QC) procedures, individual RNA-seq libraries were pooled based on their respective sample-specific 6-bp (base pairs) adaptors and paired-end sequenced at 150 bp/sequence reads (PE150) using an Illumina HiSeq 2500 sequencer.

#### Whole-genome bisulfite sequencing (WGBS)

All experiments were carried out following published procedures [56–58]. Briefly, DNA from REPC culture was isolated by phenol/chloroform extraction. DNA (100 ng) was bisulfite-converted and subjected to library preparation using the Pico Methyl-Seq™ Library Prep Kit (Zymo) following the instructions of the supplier. High-sensitivity DNA chips were used to assess libraries for quality on the Agilent Bioanalyzer and quantified with Qubit fluorometer. Libraries were sequenced on an Illumina HiSeq2500 (150-bp paired-end sequencing).

### Bioinformatics analyses for all epigenomic marks, RNA-seq, and DNA methylation

We removed raw reads that failed Illumina’s quality filter. In the REPC study, we generated a total of 385,544,396 and 428,908,598 clean paired-end reads for four ATAC-seq data sets and ten ChIP-seq data sets, respectively, using Illumina NextSeq 500. We also generated a total of 39,941,058 paired-end clean reads as the random background input. For the remaining three studies, we generated a total of 731,245,394 paired-end clean reads, and 3,247,857 and 5,709,815 paired-end clean reads as the random background input for the rumen tissue and MDBK studies, respectively. We then mapped clean reads to the cattle reference genome (UMD3.1.1) using the BWA algorithm with default settings [59]. We only kept reads uniquely aligned with less than two mismatches for the subsequent analysis. We employed MACS2.1.1 for peak calling with default parameter settings by looking for significant enrichment in the studied samples when compared to the input data file (i.e., random background) [60]. We calculated peak correlations among all 38 epigenomic samples using the following strategy. Briefly, we computed the correlation of sample A with sample B as the number of peaks in A overlapped with B, divided by the total number of peaks in A, while the correlation of B with A as the number of peaks in B overlapped with A, divided by the total number of peaks in sample B.

We employed a multivariate Hidden Markov Model (HMM), implemented in ChromHMM version 1.18 [61], to define 15 chromatin states using 200-bp sliding windows through combining all six epigenomic marks and one input random background in REPC. This method could provide an unbiased and systematic chromatin state discovery along the whole genome [13, 61]. We computed the enrichment fold of each state for each external annotation (e.g., CpG islands) as (*C*/*A*)/(*B*/*D*), where *A* is the number of bases in the chromatin state, *B* is the number of bases in the external annotation, *C* is the number of bases overlapped between state and the external annotation, and *D* is the number of bases in the genome. We calculated the significance of enrichment using Fisher’s exact test.

For all 12 RNA-seq and WGBS data sets in the REPC study (three biological replicates in each condition), we did quality control and trimming by employing FastQC (https://www.bioinformatics.babraham.ac.uk/projects/fastqc/) and Trim_Galore (version 0.4.1) (https://www.bioinformatics.babraham.ac.uk/projects/trim_galore/), respectively. Generally, we removed adapters and reads with low quality (*Q* < 20) or shorter than 20 bp. For RNA-seq, we used STAR aligner [62] and Cufflinks software tools [63] to quantify gene expression and conduct differentially expression analysis, where only the uniquely mapped reads were used. We used the FPKM value of each gene as its normalized expression level. We defined DEGs as Bonferroni-corrected *P* value less than 0.05 and log_2_(fold change) greater than 2. For WGBS, all clean data were mapped to the cattle reference genome (UMD 3.1.1) using bowtie2 [64]. We then applied Bismark software [65] with default settings to extract methylcytosine information. We kept loci with at least 10 clean reads coverage for further analyses. We determined DMRs using methylKit with 500-bp window size and 500-bp step size [66]. Briefly, we used a logistic regression model, implemented in the *calculateDiffMeth* function, to detect DMRs. We computed *P* values by comparing the model fitness of alternative models (with treatment effects) to the null model (without treatment effects) and corrected to *q* values for multiple testing using the SLIM method [67]. We considered *q* value less than 0.05 and the absolute value of the difference in methylation greater than 10% as DMRs.

### GWAS signal enrichment analysis

We applied a sum-based marker set test, implemented by the R package for Quantitative Genetic and Genomic analyses (QGG package; http://psoerensen.github.io/qgg/), for GWAS signal enrichment analyses across all 15 chromatin states and butyrate-induced DEGs. Previous studies demonstrated that this approach has equal or better power than other commonly used marker set tests, particularly in highly polygenic complex phenotypes [23, 24, 68–70]. Briefly, we calculated the following summary statistics for each genomic feature (e.g., a chromatin state or a list of DEGs):

$$ {T}_{\mathrm{sum}}={\sum}_{i=1}^{m_{\mathrm{f}}}{b}^2, $$

where *T*_sum_ is the summary statistics for each genomic feature, *b* is the SNP effect in the single-marker GWAS; *b*^2^ is the square of *b*, and *m*_f_ is the number of SNPs overlapped a genomic feature being tested. We determined the association of a genomic feature with a complex trait by a 10,000-times circular-genotype permutation test for *T*_sum_ of the genomic feature. We calculated an empirical *P* value for the genomic feature as the proportion of random *T*_sum_ from permutation greater than the observed *T*_sum_. In total, we analyzed 45 complex traits, including 18 body conformation, 6 milk production, 12 reproduction, 8 health, and 1 feed efficiency. The details of the signal-marker GWAS analyses (imputed sequence marker; *n* = ~ 3 million) for body conformation, reproduction, and milk production traits from 27,214 US Holstein bulls could be found in [71]. The details of health traits (imputed sequence marker; *n* = ~ 3 million) for ~ 10,000 bulls could be found in Freebern et al. (2019, submitted), while the details of feed efficiency (high-density marker; *n* = ~ 300,000) for 3947 Holstein cows (i.e., residual feed intake, RFI) were described by Li et al. (2019, accepted in J Dairy Sci).

### Tissue enrichment analysis for DEGs and other down-stream bioinformatics analysis

To detect tissue/cell types that may be associated with DEGs induced by butyrate treatment, we conducted enrichment analyses for these DEGs using tissue/cell type-specific genes. We previously uniformly analyzed a total of 732 RNA-seq data sets to detect tissue/cell type-specific genes while accounting for known covariates (e.g., sex and age), including 91 different tissue/cell types in cattle. The details of the tissue/cell type-specific genes were summarized by Fang et al. (2019; submitted; https://github.com/LingzhaoFang1/Cattle-GeneAtlas). For tissue/cell type-specific genes, we chose the top 5% of genes that were specifically highly expressed in a tissue/cell type as the corresponding tissue/cell type-specific genes. We then employed a hypergeometric test, similar to GO enrichment analysis implemented in clusterProfiler [72]. For exploring the biological function of a list of genes, we conducted the gene functional enrichment analysis using R package clusterProfiler [72], where a hypergeometric test, based on the current GO and KEGG databases, was employed. We used HOMER (http://homer.ucsd.edu/homer/motif/) to conduct the motif enrichment analysis for chromatin states considering the whole genome as background. We adjusted *P* values for multiple testing using the FDR method.

## Additional files

Additional file 1: **Figure S1.** General characteristics for 38 epigenomic data sets. **Figure S2.** Distribution of peak-length for all 38 epigenomic data sets. **Figure S3.** Distribution of distances between peaks and their nearest genes for all 38 epigenomic data sets. **Figure S4.** Correlations of peak-length vs. chromosome-length for all 38 epigenomic data sets. **Figure S5.** Correlations of peak-length vs. gene-length for all 38 epigenomic data sets. **Figure S6.** Correlations of peak-length vs. exon-length for all 38 epigenomic data sets. **Figure S7.** Correlations of epigenomic, RNA-seq, and DNA methylation data sets. **Figure S8.** Genes specifically highly expressed (*n* = 1230; top 5%) in rumen epithelial primary cells (REPC). **Figure S9.** Gene-length distribution across the four gene sets. **Figure S10.** The dn/ds ratio comparison for the four gene sets corresponding to orthologous genes across mammals. **Figure S11.** The expression levels of the four orthologous gene sets in 53 human tissues. **Figure S12. **The expression levels of the four orthologous gene sets across 159 mouse tissues. **Figure S13.** The expression levels of the four orthologous gene sets in 174 sheep tissues. **Figure S14.** Functional enrichment analysis for the four gene sets. **Figure S15.** Enrichment of chromatin states for differentially methylated regions induced by butyrate treatment in rumen epithelial primary cells (REPC). **Figure S16.** Butyrate-induced dynamics in chromatin states and gene expression. **Figure S17.** Associations of downregulated differentially expressed genes (down-DEGs) with alterations of chromatin states. (DOCX 6756 kb)

Additional file 2: **Table S1.** Mapping summary of all the epigenome, RNA-seq, and DNA methylation data sets. (XLSX 17 kb)

Additional file 3: **Table S2.** Motif enrichment analysis for 13 chromatin states (excluding repressed polycomb and quiescent states). (XLSX 730 kb)

Additional file 4: **Table S3.** Summary of upregulated differentially expressed genes (DEGs) detected in rumen epithelial primary cells (REPC) before and after (24 h) butyrate treatment. (XLSX 131 kb)

Additional file 5: **Table S4.** Summary of downregulated differentially expressed genes (DEGs) detected in rumen epithelial primary cells (REPC) before and after (24 h) butyrate treatment. (XLSX 70 kb)

Additional file 6: **Table S5.** Enrichment analysis of differentially expressed genes (DEGs) for tissue-specific genes across 91 tissues/cell types in cattle. (XLSX 47 kb)

Additional file 7: **Table S6.** Significantly differentially methylated regions in rumen epithelial primary cells (REPC) before and after (24 h) butyrate treatment. (XLSX 12 kb)

## Data Availability

All high-throughput sequencing data analyzed in this study are deposited in NCBI GEO database under accession number GSE129423 [73]. The annotated chromatin states of REPC and all the peaks of epigenomic marks in this study are publicly available at [74]. All the data will be deposited in the FAANG portal in the near future and be available to FAANG project community. The reference genome and gene annotation files (including all the sequence ontology, orthologues genes among mammals, and evolutionarily conserved regions) of UMD3.1.1 were downloaded from Ensembl v94 [75]. The Cattle QTLdb (release 37, Dec. 23, 2018) was obtained from [34]. The gene expression among 53 tissues in human was obtained from [76]. The gene expression among 153 tissues in mouse was downloaded from [77]. The gene expression among 174 tissues in sheep was downloaded from [78]. The transcriptional factors in cattle were obtained from [30]. The selection signatures in cattle were obtained from [37]. The eQTLs of muscle in cattle were obtained from [36].

## Abbreviations

BivFlnk: Flanking bivalent TSS/enhancers
DEG: Differentially expressed genes
DMR: Differentially methylated regions
EnhA: Active enhancer
EnhAATAC: Active enhancer with ATAC
EnhWk: Weak active enhancer
eQTL: Expression quantitative trait loci
HDAC: Histone deacetylase
MDBK: Madin-Darby bovine kidney epithelial cells
REPC: Rumen epithelial primary cells
REPC-SG: REPC-specific genes
ReprPC: Repressive Polycomb
TES: Transcriptional end sites
TSS: Transcriptional start sites
TssA: Active TSS
TssAFlnk: Flanking active TSS
TxFlnk: Transcribed at gene 5′ and 3′
VFA: Volatile fatty acids
WGBS: Whole-genome bisulfite sequencing
