# Supplementary material for: Functional annotation of the cattle genome through systematic discovery and characterization of chromatin states and butyrate-induced variations
Source: BMC Biol. 2019 Aug 16;17:68. doi: 10.1186/s12915-019-0687-8 (PMC6698049; doi:10.1186/s12915-019-0687-8)
Supplement: Supplementary file 1 — Figure S1. General characteristics for 38 epigenomic data sets. Figure S2. Distribution of peak-length for all 38 epigenomic data sets. Figure S3. Distribution of distances between peaks and their nearest genes for all 38 epigenomic data sets. Figure S4. Correlations of peak-length vs. chromosome-length for all 38 epigenomic data sets. Figure S5. Correlations of peak-length vs. gene-length for all 38 epigenomic data sets. Figure S6. Correlations of peak-length vs. exon-length for all 38 epigenomic data sets. Figure S7. Correlations of epigenomic, RNA-seq, and DNA methylation data sets. Figure S8. Genes specifically highly expressed (n = 1230; top 5%) in rumen epithelial primary cells (REPC). Figure S9. Gene-length distribution across the four gene sets. Figure S10. The dn/ds ratio comparison for the four gene sets corresponding to orthologous genes across mammals. Figure S11. The expression levels of the four orthologous gene sets in 53 human tissues. Figure S12. The expression levels of the four orthologous gene sets across 159 mouse tissues. Figure S13. The expression levels of the four orthologous gene sets in 174 sheep tissues. Figure S14. Functional enrichment analysis for the four gene sets. Figure S15. Enrichment of chromatin states for differentially methylated regions induced by butyrate treatment in rumen epithelial primary cells (REPC). Figure S16. Butyrate-induced dynamics in chromatin states and gene expression. Figure S17. Associations of downregulated differentially expressed genes (down-DEGs) with alterations of chromatin states. (DOCX 6756 kb) [file 12915_2019_687_MOESM1_ESM.docx]

**
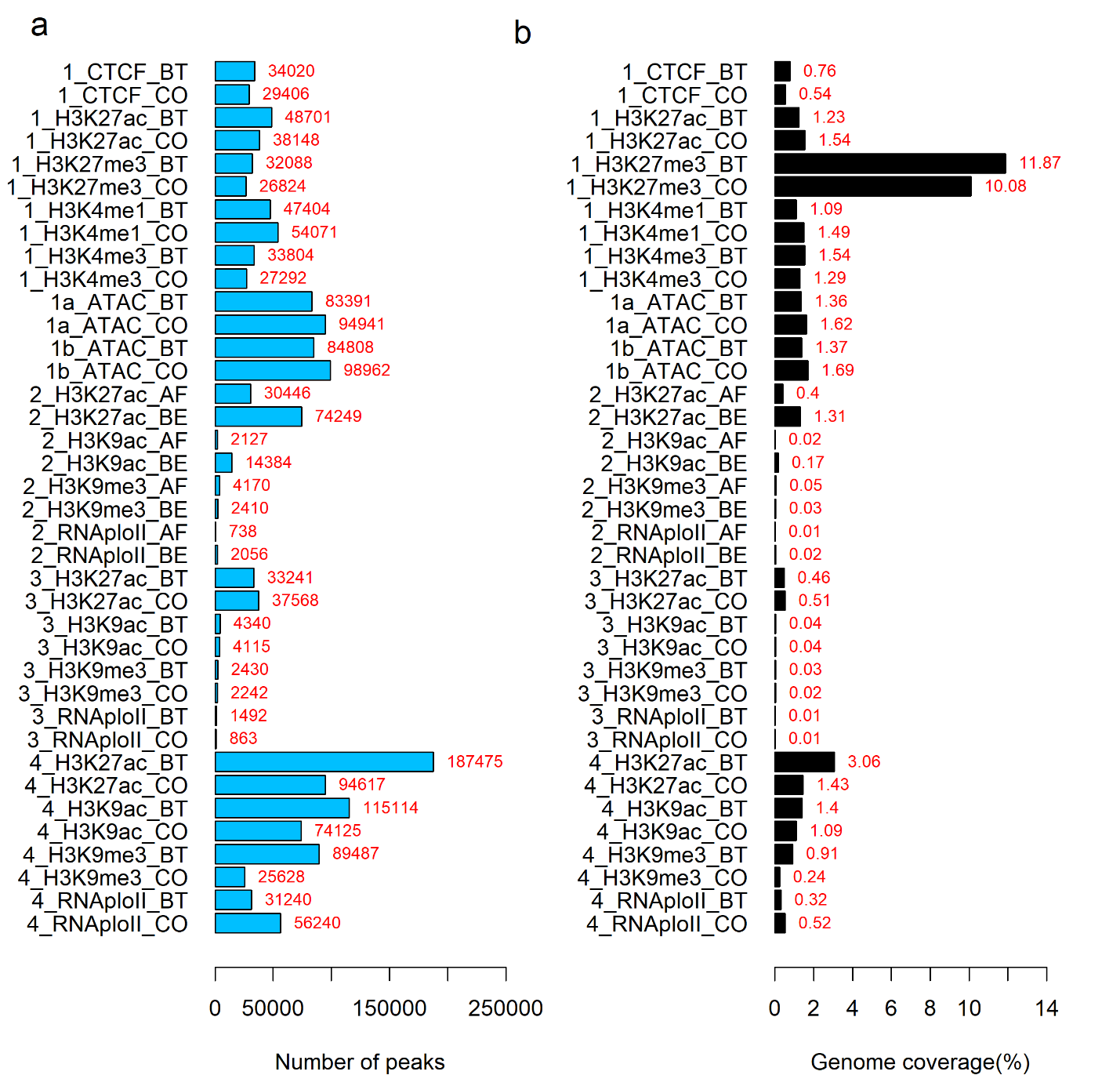
**

**Figure S1. General characteristics for the 38 epigenomic data sets. a** Peak numbers of each sample across the four experiments. **b** Genomic coverage percentage of peaks for each sample across the four experiments. CO and BT represent the control and butyrate-treatment group, respectively, while BE and AF represent before and after weaning, respectively.

**
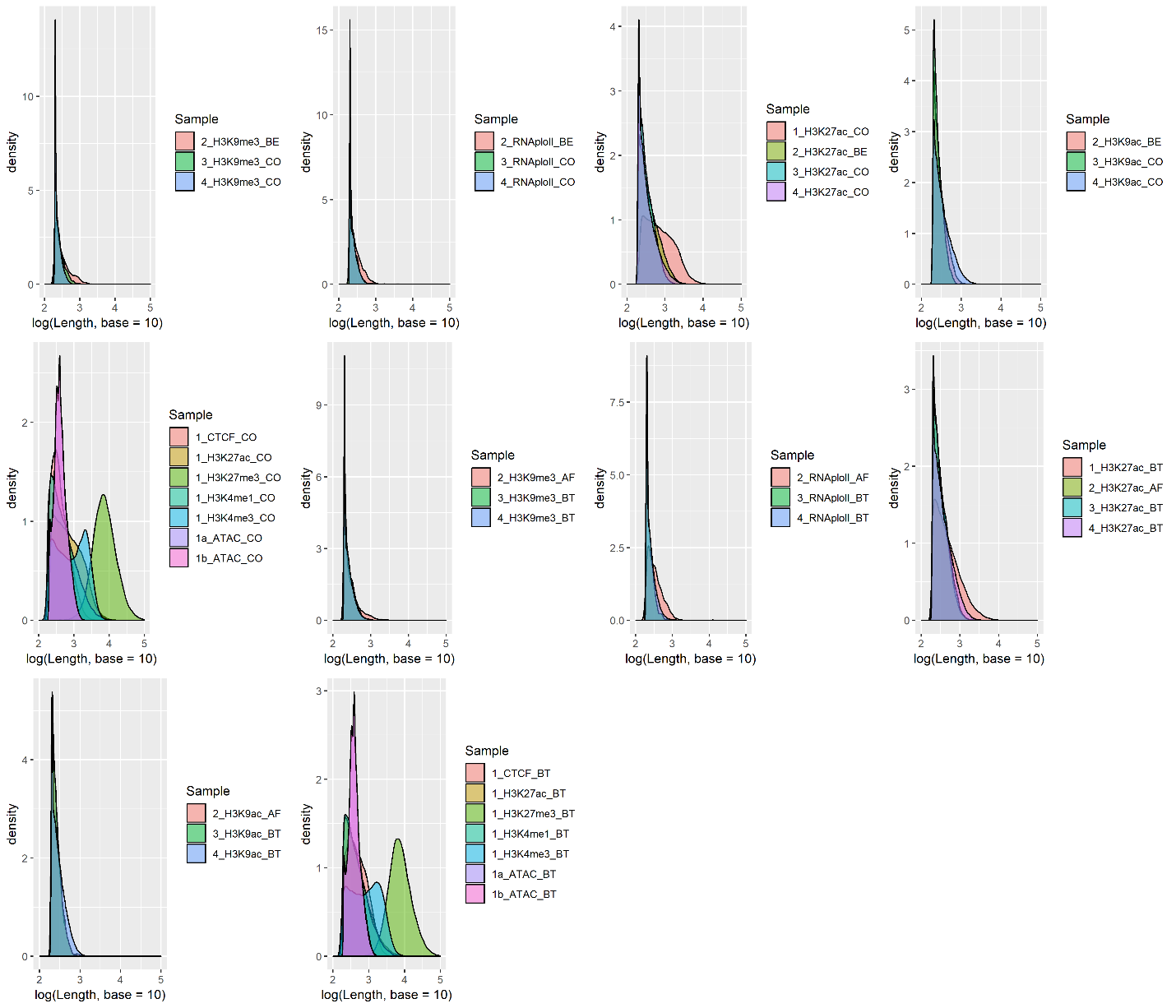
**

**Figure S2. Distribution of peak-length for all the 38 epigenomic data sets across the four experiments.** CO and BT represent the control and butyrate-treatment group, respectively, while BE and AF represent before and after weaning, respectively. ATAC-seq has two biological replicates (a, b).

**
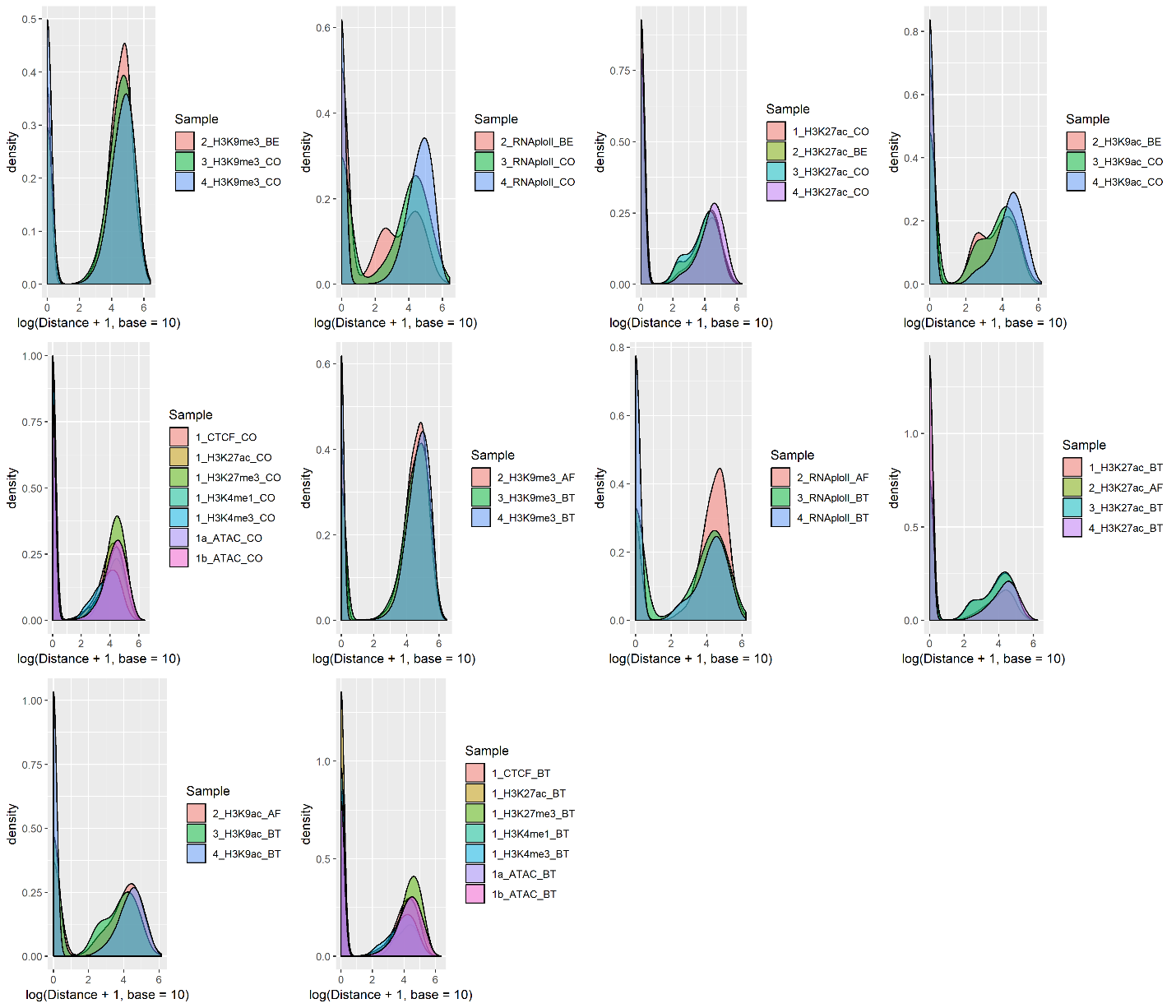
**

**Figure S3. Distribution of distances between peaks and their nearest genes for all the 38 epigenomic data sets across the four experiments.** CO and BT represent the control and butyrate-treatment group, respectively, while BE and AF represent before and after weaning, respectively. ATAC-seq has two biological replicates (a, b).

**
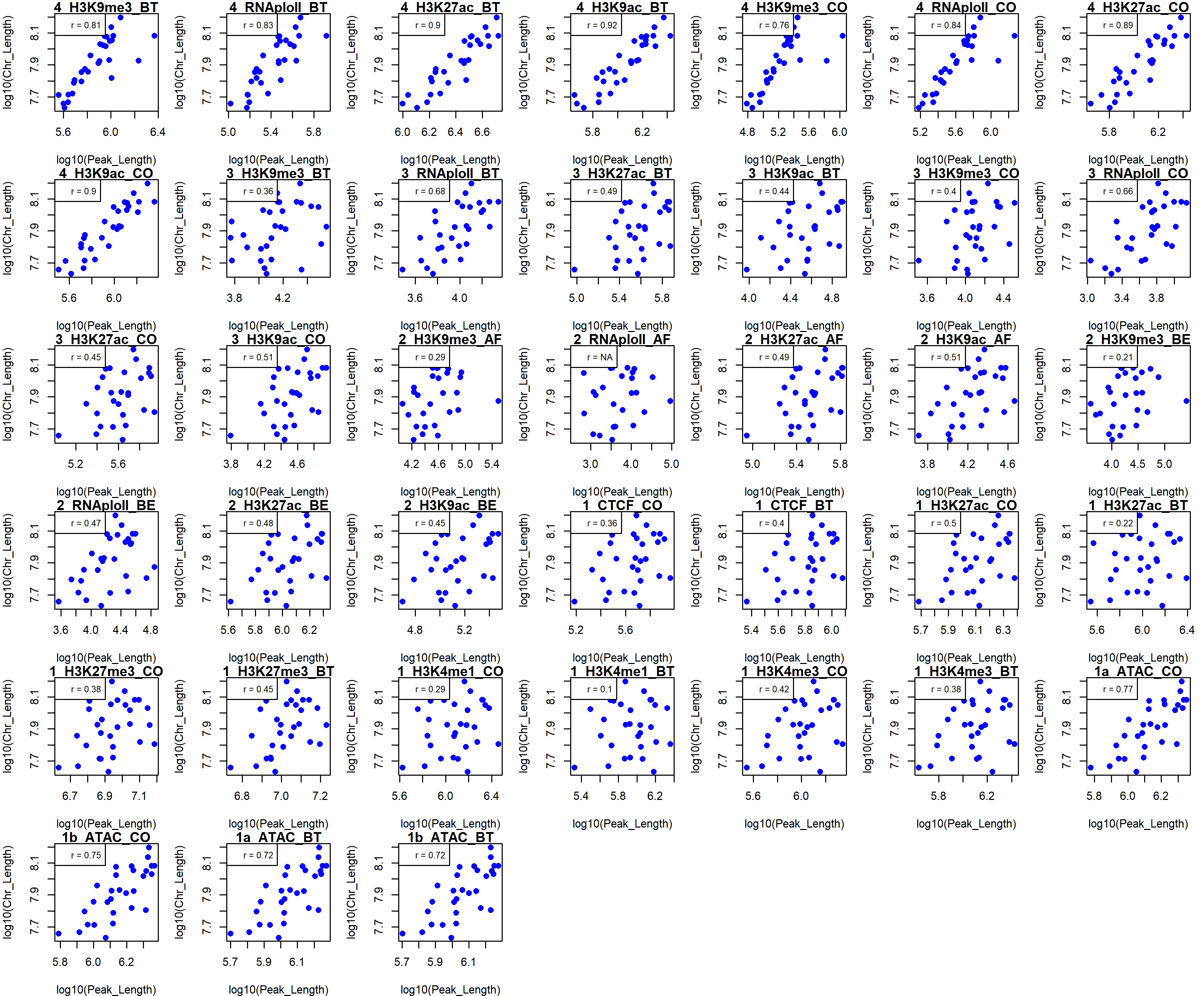
**

**Figure S4. Correlations of chromosome-wide peak-length vs. chromosome-length for all the 38 epigenomic data sets across the four experiments.** CO and BT represent the control and butyrate-treatment group, respectively, while BE and AF represent before and after weaning, respectively. ATAC-seq has two biological replicates (a, b).


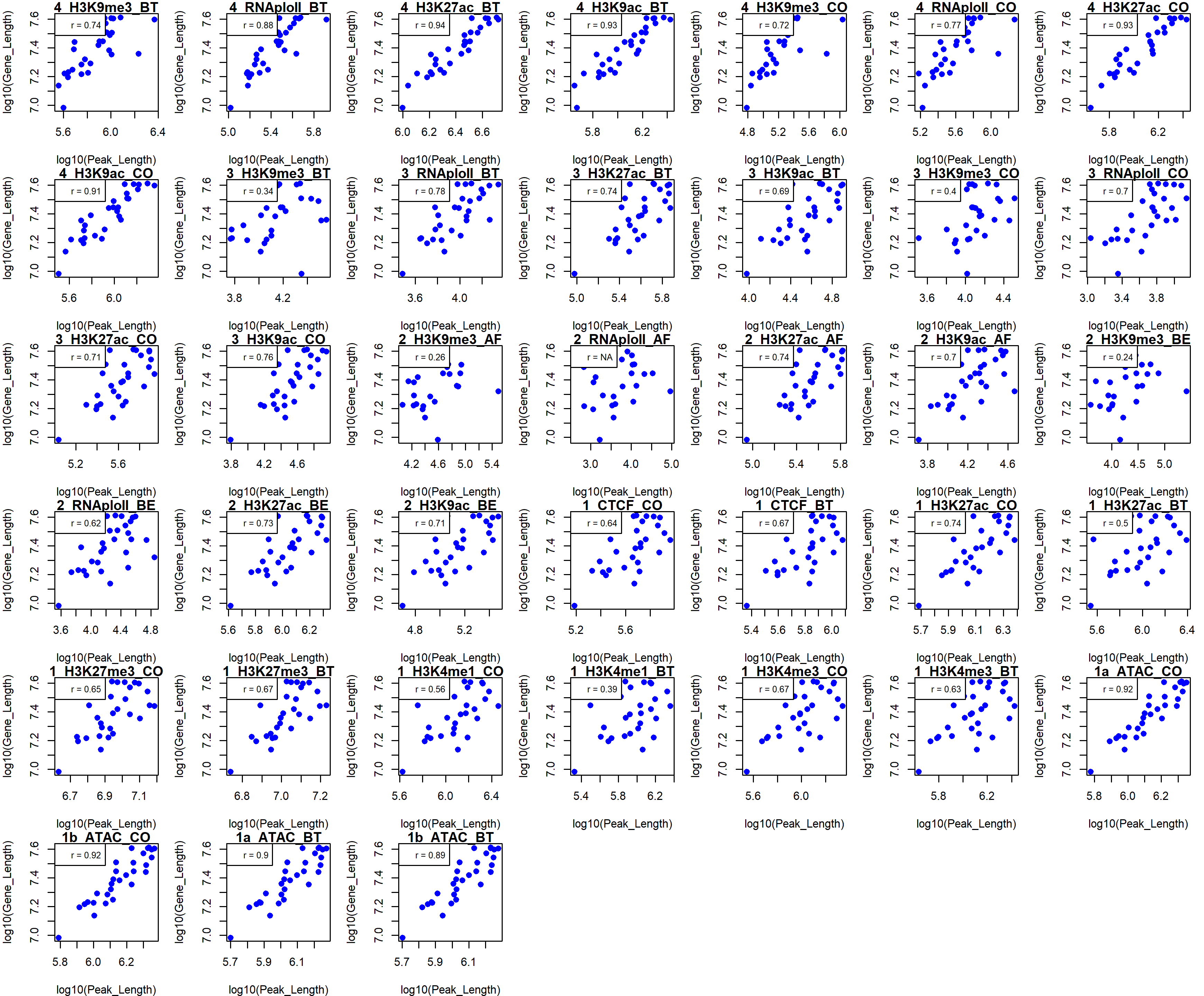


**Figure S5. Correlations of peak-length and gene-length for all the 38 epigenomic data sets across the four experiments.** CO and BT represent the control and butyrate-treatment group, respectively, while BE and AF represent before and after weaning, respectively. ATAC-seq has two biological replicates (a, b).


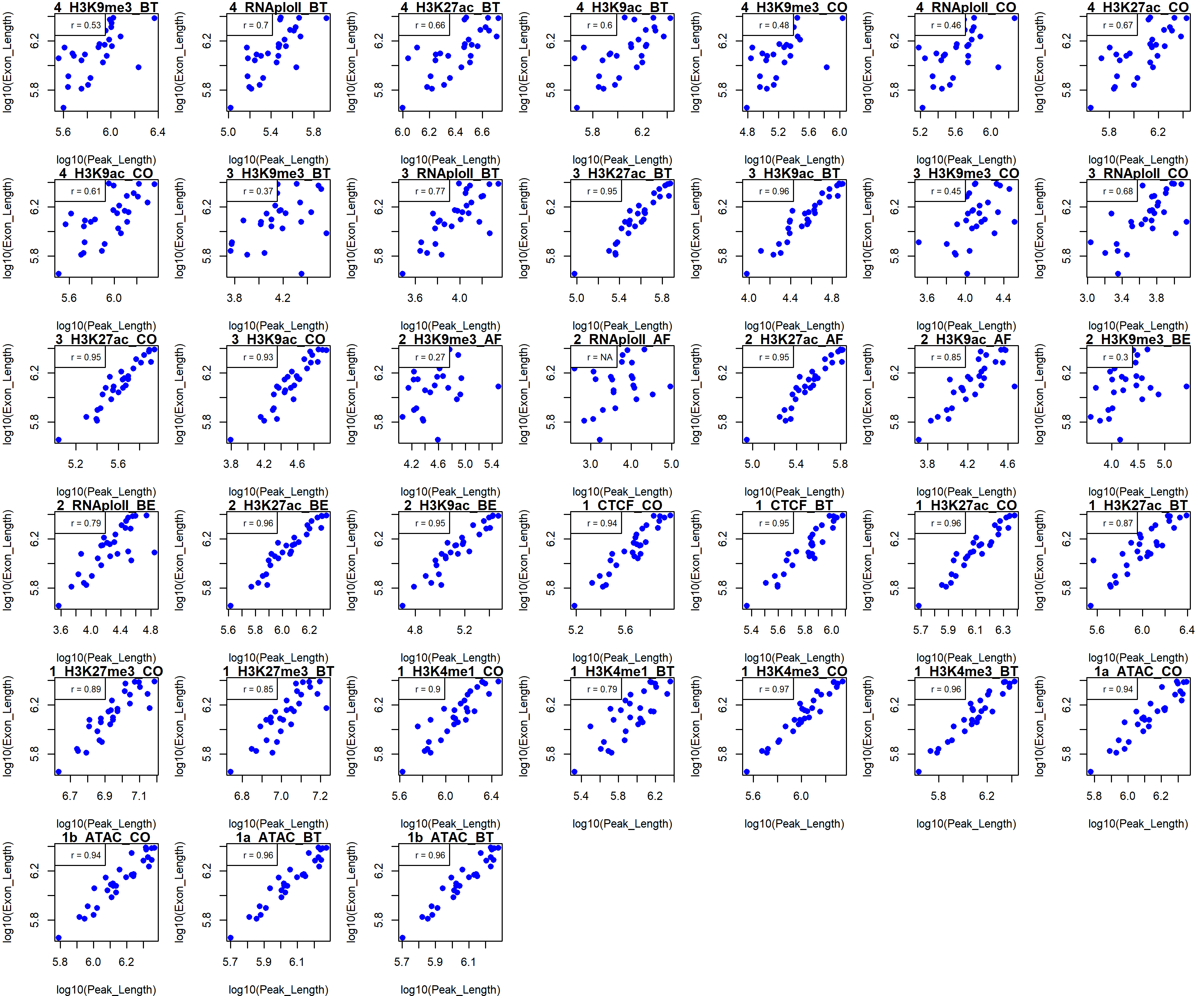


**Figure S6. Correlations of peak-length vs. exon-length chromosome-wide for all the 38 epigenomic data sets across the four experiments.** CO and BT represent the control and butyrate-treatment group, respectively, while BE and AF represent before and after weaning, respectively. ATAC-seq has two biological replicates (a, b).


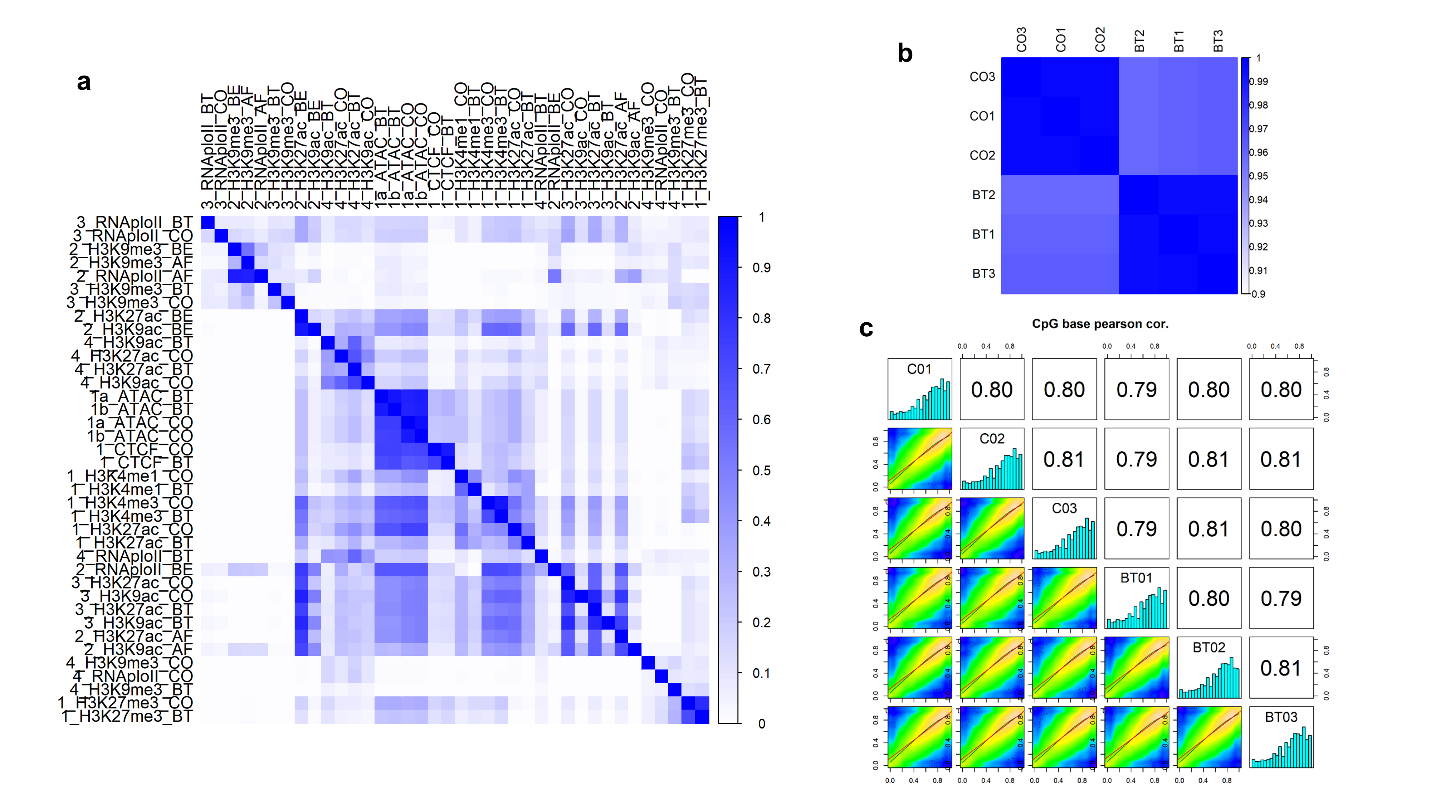


**Figure S7. Correlations of epigenomic, RNA-seq and DNA methylation data sets.** CO and BT represent the control and butyrate-treatment group, respectively, while BE and AF represent before and after weaning, respectively. ATAC-seq has two biological replicates (a, b). **a** Correlations among epigenomic data sets across the four experiments. **b** Gene expression correlations (Pearson’s Correlation) among the six samples in Rumen Epithelial Primary Cells before (CO) and after (BT; 24-h) butyrate treatment. **c** DNA methylation correlations among the six samples in Rumen Epithelial Primary Cells before (CO) and after (BT; 24-h) butyrate treatment.


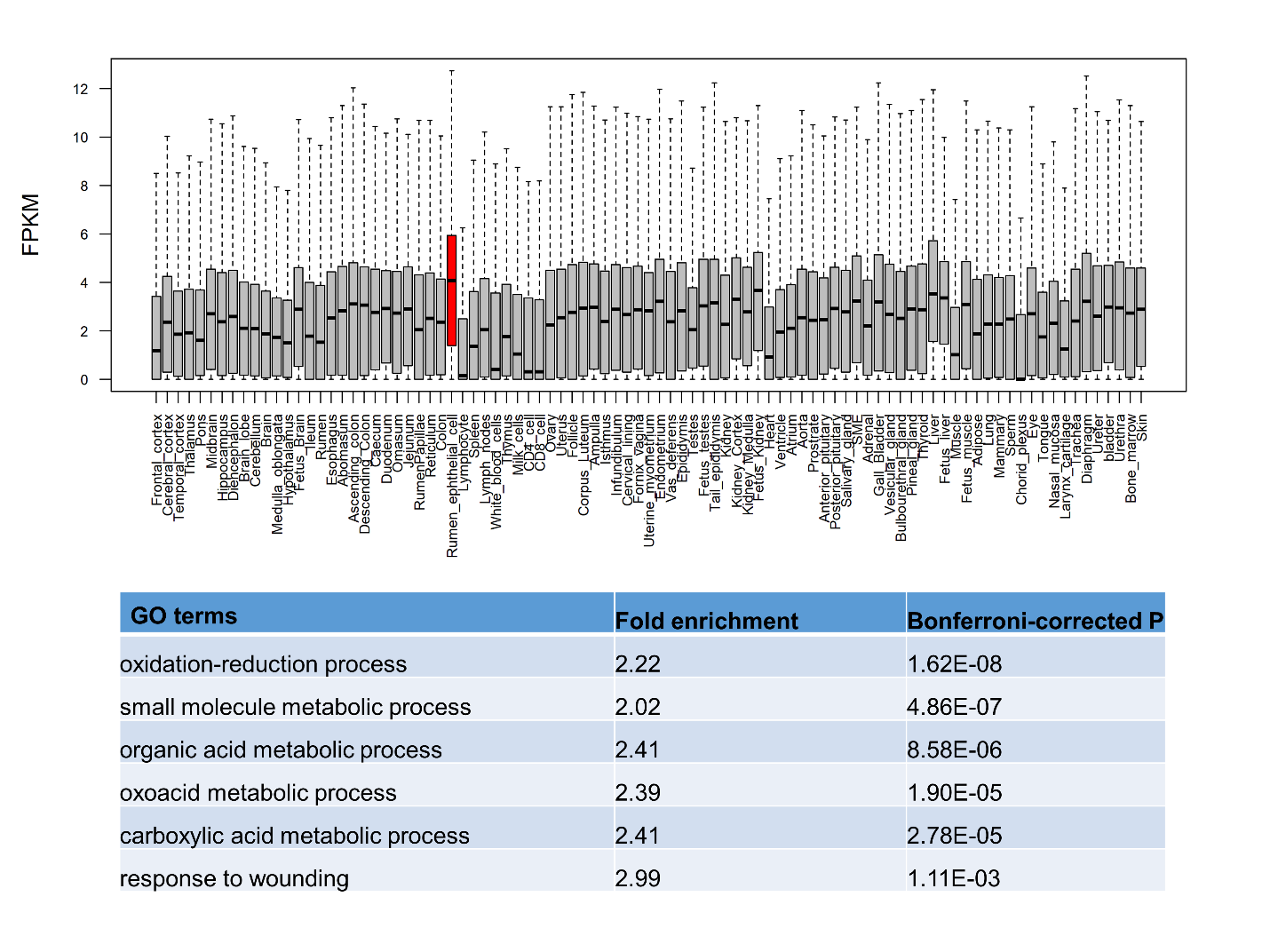


**Figure S8. Genes specifically highly expressed (n = 1230; top 5%) in Rumen Epithelial Primary Cells (REPC). Up figure** Expression of the REPC-specific genes across 91 tissues and cell types. **Down figure** Significantly enriched Gene Ontology terms for REPC-specific genes.


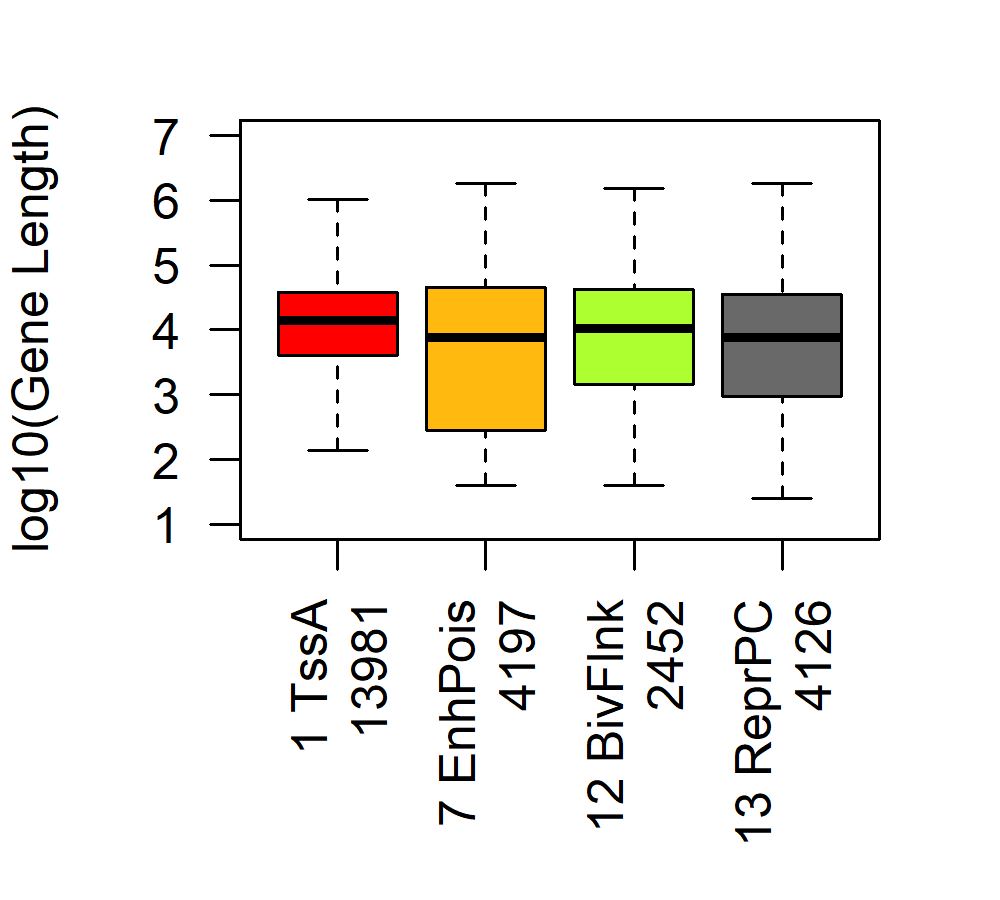


**Figure S9. Genes-length distribution across the four gene sets.** Genes with active promoters (TssA; n = 13,981), genes with poised enhancers but not active promoters (EnhPois; n = 4,197), genes with flanking bivalent TSS/enhance but not active promoters (BivFlnk; n = 2,452), and genes with repressive Polycomb but not active promoters (ReprPC; n = 4,126).


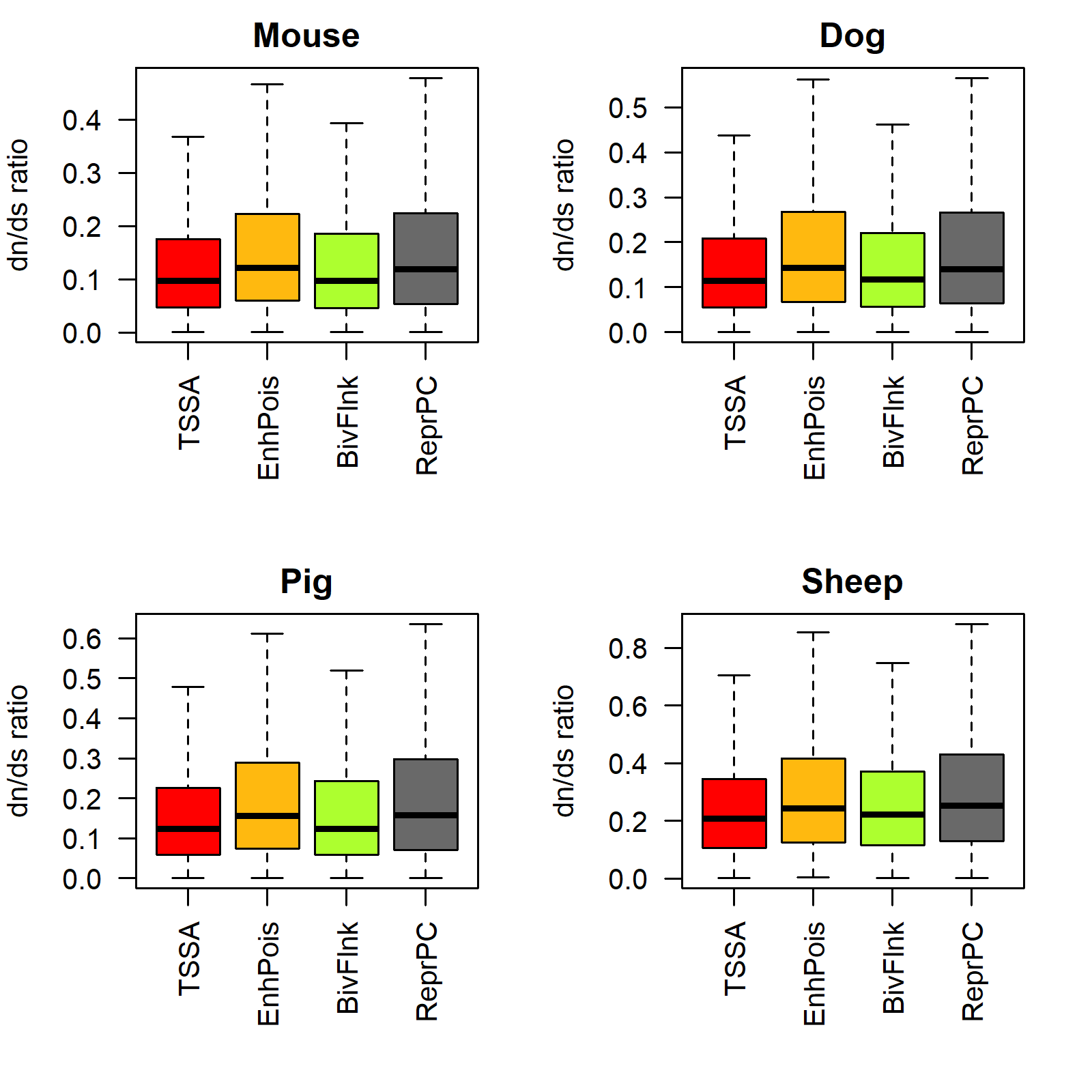


**Figure S10. The dn/ds ratio comparison for the four gene sets corresponding to orthologous genes across mammals.** Genes with active promoters (TssA), genes with poised enhancers but not active promoters (EnhPois), genes with flanking bivalent TSS/enhance but not active promoters (BivFlnk), and genes with repressive Polycomb but not active promoters (ReprPC).


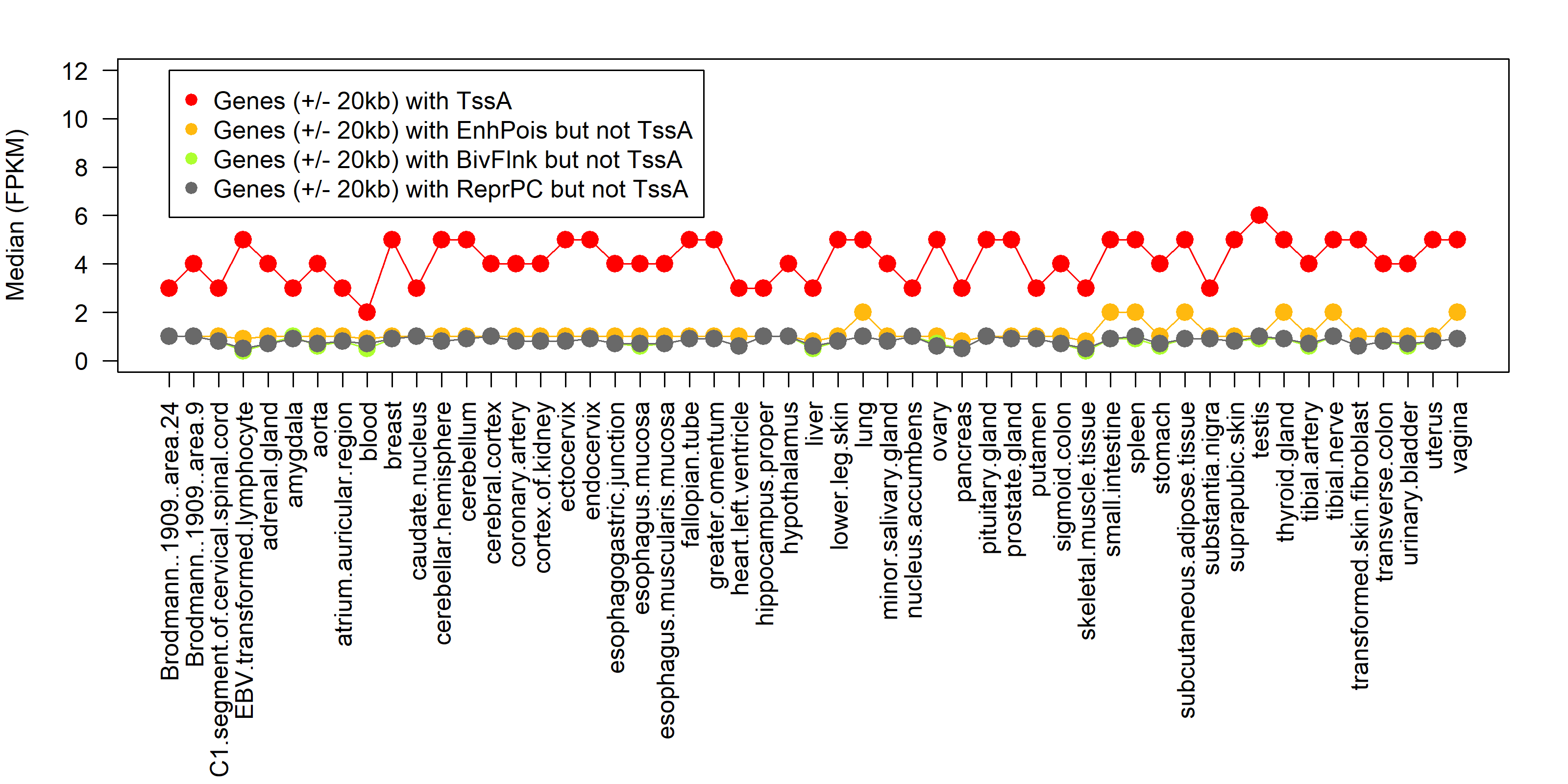


**Figure S11. The expression levels of the four orthologous-gene sets in 53 human tissues.** Genes with active promoters (TssA; n = 11,695), genes with poised enhancers but not active promoters (EnhPois; n = 2,622), genes with flanking bivalent TSS/enhance but not active promoters (BivFlnk; n = 1,932), and genes with repressive Polycomb but not active promoters (ReprPC; n = 2,972).


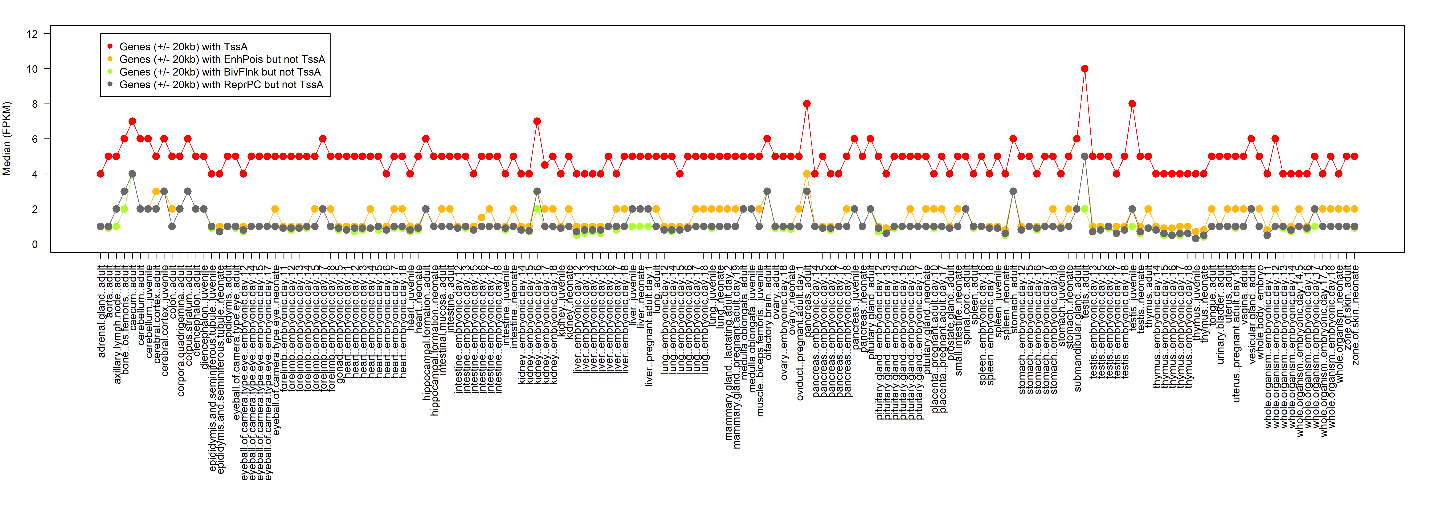


**Figure S12. The expression levels of the four orthologous-gene sets across 159 mouse tissues.** Genes with active promoters (TssA; n = 11,701), genes with poised enhancers but not active promoters (EnhPois; n = 2,587), genes with flanking bivalent TSS/enhance but not active promoters (BivFlnk; n = 1,885), and genes with repressive Polycomb but not active promoters (ReprPC; n = 2,950).


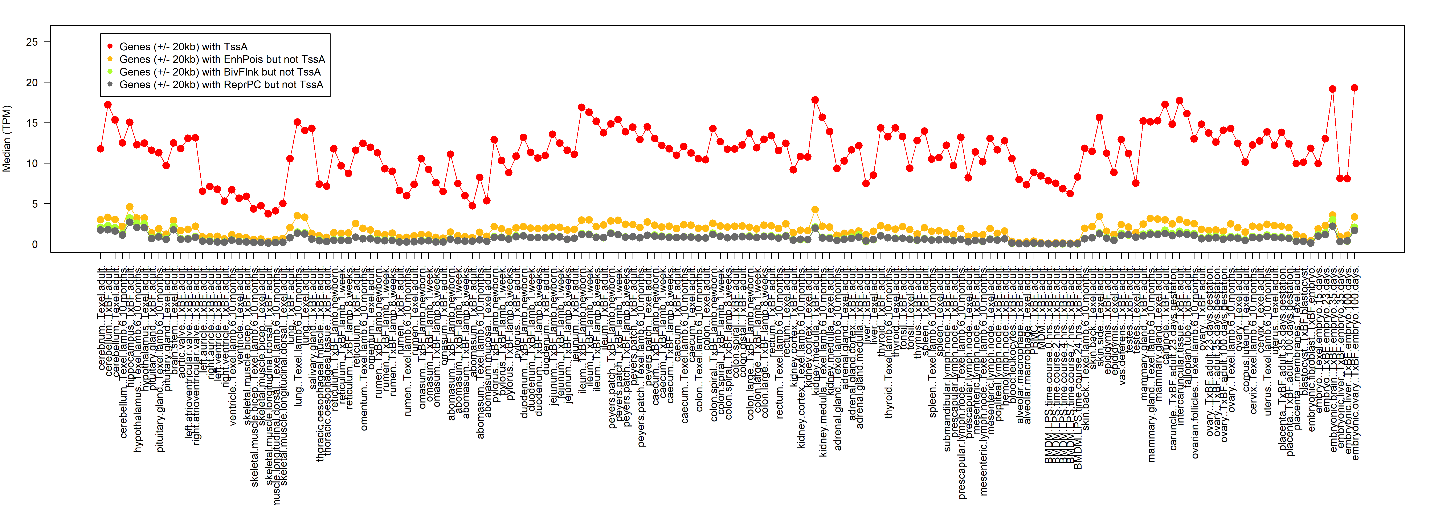


**Figure S13. The expression levels of the four orthologous-gene sets in 174 sheep tissues.** Genes with active promoters (TssA; n = 11,145), genes with poised enhancers but not active promoters (EnhPois; n = 2,621), genes with flanking bivalent TSS/enhance but not active promoters (BivFlnk; n = 1,826), and genes with repressive Polycomb but not active promoters (ReprPC; n = 2,936).


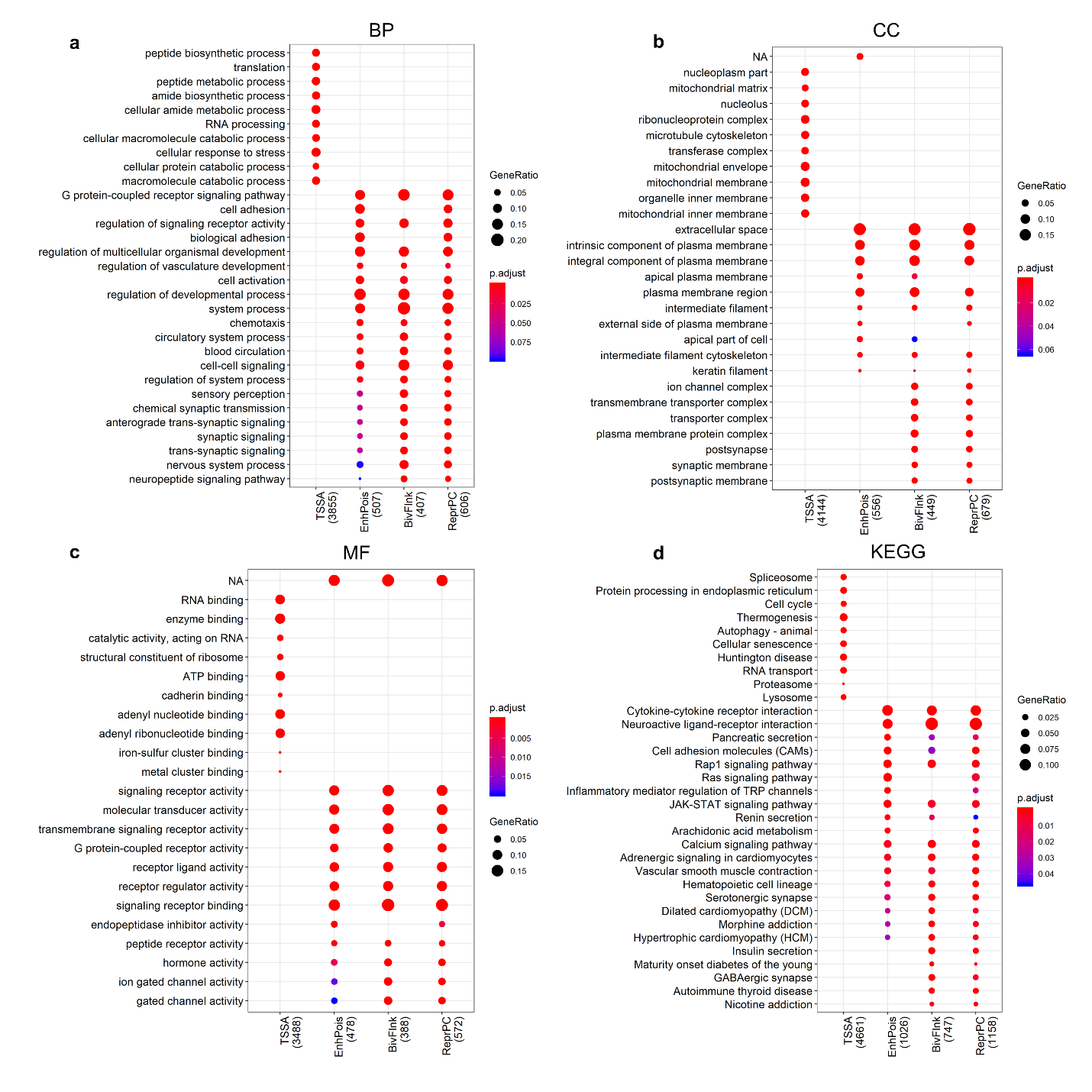


**Figure S14. Functional enrichment analysis for the four gene sets.** Genes with active promoters (TssA; n = 13,981), genes with poised enhancers but not active promoters (EnhPois; n = 4,197), genes with flanking bivalent TSS/enhance but not active promoters (BivFlnk; n = 2,452), and genes with repressive Polycomb but not active promoters (ReprPC; n = 4,126). **BP** Biological Processes in Gene Ontology database; **CC** Cellular Components in Gene Ontology database; **MF** Molecular Function in Gene Ontology database; **KEGG** Pathways in Kyoto Encyclopedia of Genes and Genomes.


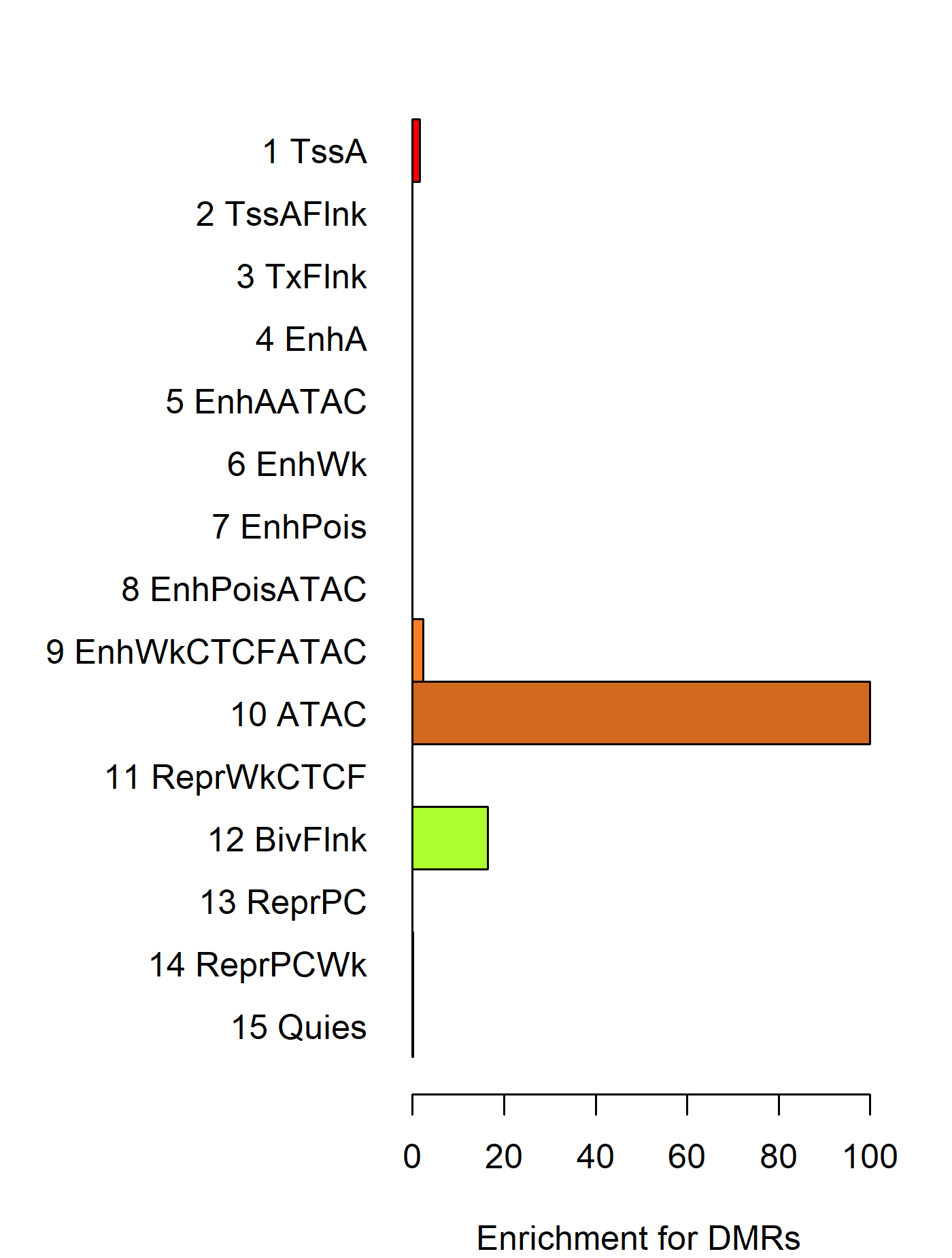


**Figure S15. Enrichment of chromatin states for differentially methylated regions induced by butyrate treatment (24h) in Rumen Epithelial Primary Cells (REPC).**


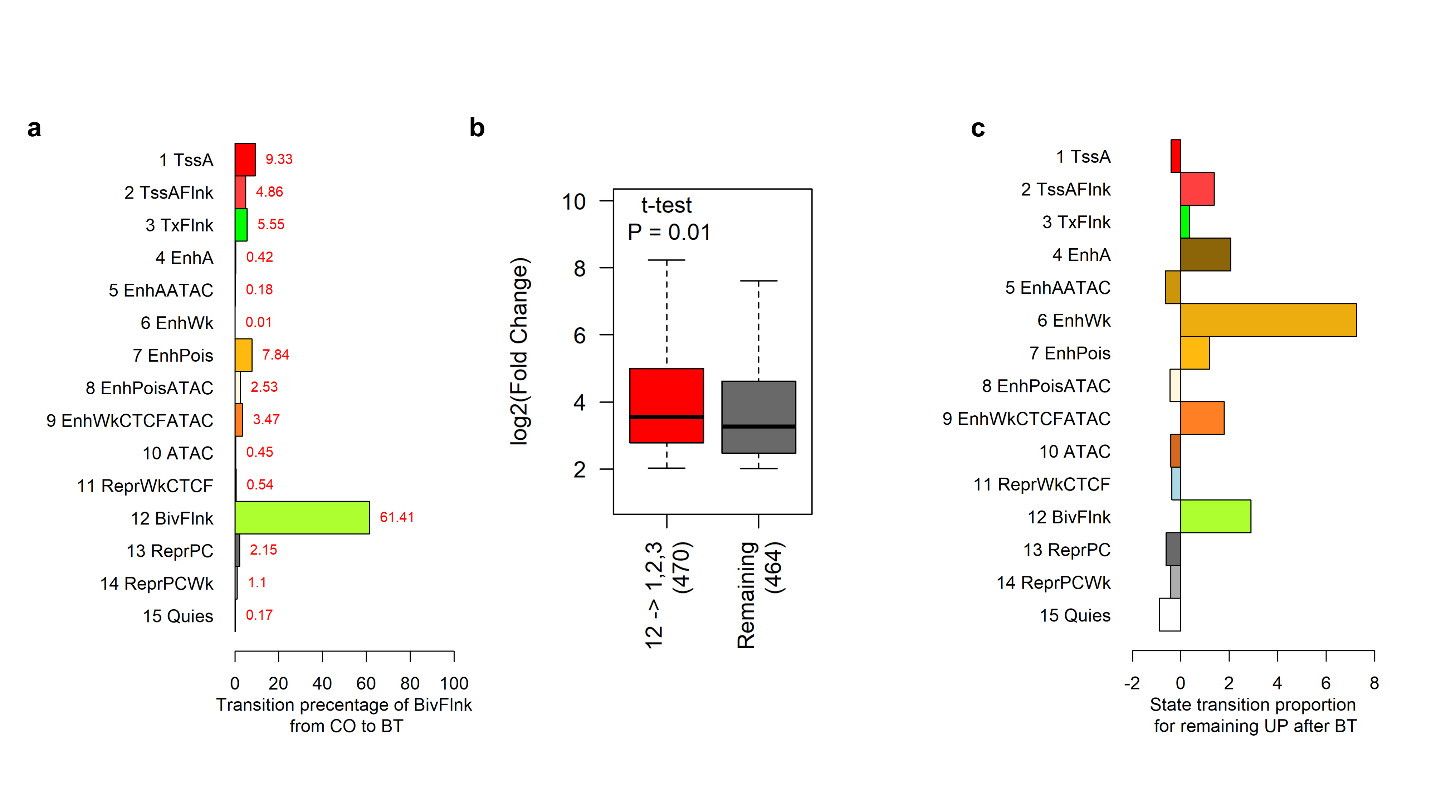


**Figure S16. Butyrate-induced dynamics in chromatin states and gene expression.** **a** The transition proportion of flanking bivalent TSS/enhancers (BivFlnk) to other chromatin states at 24h post butyrate treatment compared to the control group (before butyrate treatment). **b** Comparison of fold changes between up-regulated DEGs (n = 470) associated with the transition from BivFlnk to active promoter/transcript (TssA, TaaAFlnk and TxFlnk, 12 ­> 1, 2, 3) and the remaining up-regulated DEGs (n = 464). **c** The dynamics of chromatin states within the remaining up-regulated DEGs (± 20Kb) before and after butyrate treatment.


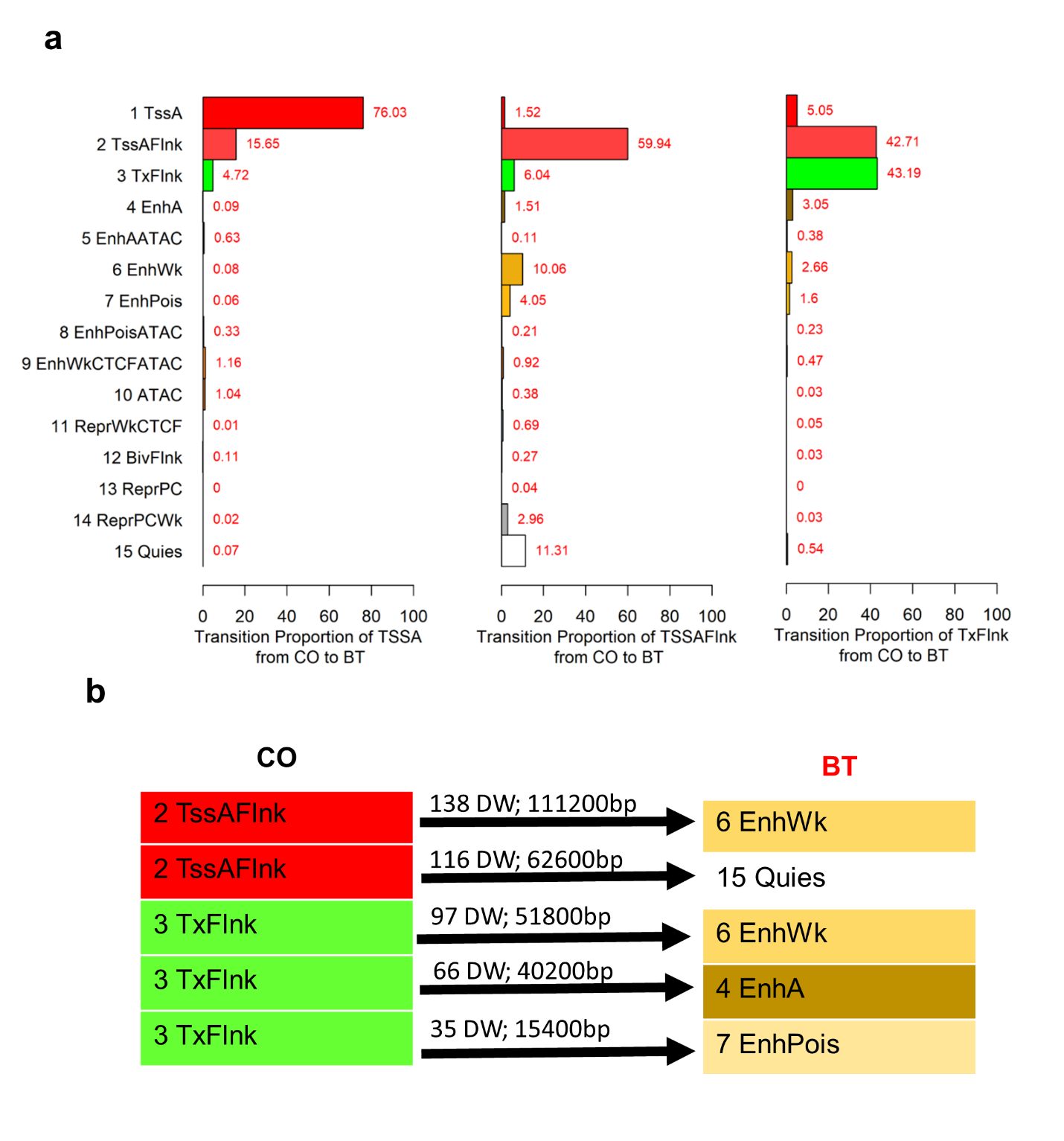


**Figure S17.** **Associations of down-regulated differentially expressed genes (down-DEGs) with alterations of chromatin states. a** The transition proportions for active TSS (TssA, left), flanking active TSS (TssAFlnk, middle), and flanking transcript (TxFlnk, right) at 24h post butyrate treatment compared to the control group (before butyrate treatment), respectively. **b** The top five transitions of chromatin states within down-regulated (DW) DEGs. The values on each arrow are the number of down-regulated genes and chromosome length involved in the particular state-transition, respectively.
